# The ESCRT-III molecules regulate the apical targeting of bile salt export pump

**DOI:** 10.1186/s12929-020-00706-2

**Published:** 2021-03-09

**Authors:** Shang-Hsin Wu, Mei-Hwei Chang, Ya-Hui Chen, Hui-Lin Wu, Huey-Huey Chua, Chin-Sung Chien, Yen-Hsuan Ni, Hui-Ling Chen, Huey-Ling Chen

**Affiliations:** 1 Graduate Institute of Clinical Medicine, National Taiwan University College of Medicine, Taipei, 100 Taiwan; 2 Department of Pediatrics, National Taiwan University College of Medicine and National Taiwan University Children’s Hospital, Taipei, 100 Taiwan; 3 Hepatitis Research Center, National Taiwan University Hospital, Taipei, 100 Taiwan; 4 Medical Microbiota Center of the First Core Laboratory, National Taiwan University College of Medicine, Taipei, 100 Taiwan; 5 Department and Graduate Institute of Medical Education and Bioethics, National Taiwan University College of Medicine, Taipei, 100 Taiwan

**Keywords:** Apical trafficking, Bile salt export pump, Cholestasis, CHMP5, ESCRT, Liver development, Post-Golgi trafficking, Subapical compartment, Rab11

## Abstract

**Background:**

The bile salt export pump (BSEP) is a pivotal apical/canalicular bile salt transporter in hepatocytes that drives the bile flow. Defects in BSEP function and canalicular expression could lead to a spectrum of cholestatic liver diseases. One prominent manifestation of BSEP-associated cholestasis is the defective canalicular localization and cytoplasmic retention of BSEP. However, the etiology of impaired BSEP targeting to the canalicular membrane is not fully understood. Our goal was to discover what molecule could interact with BSEP and affect its post-Golgi sorting.

**Methods:**

The human BSEP amino acids (a.a.) 491-630 was used as bait to screen a human fetal liver cDNA library through yeast two-hybrid system. We identified a BSEP-interacting candidate and showed the interaction and colocalization in the co-immunoprecipitation in hepatoma cell lines and histological staining in human liver samples. Temperature shift assays were used to study the post-Golgi trafficking of BSEP. We further determine the functional impacts of the BSEP-interacting candidate on BSEP in vitro. A hydrodynamically injected mouse model was established for in vivo characterizing the long-term impacts on BSEP.

**Results:**

We identified that charged multivesicular body protein 5 (CHMP5), a molecule of the endosomal protein complex required for transport subcomplex-III (ESCRT-III), interacted and co-localized with BSEP in the subapical compartments (SACs) in developing human livers. Cholestatic BSEP mutations in the CHMP5-interaction region have defects in canalicular targeting and aberrant retention at the SACs. Post-Golgi delivery of BSEP and bile acid secretion were impaired in ESCRT-III perturbation or CHMP5-knockdown hepatic cellular and mouse models. This ESCRT-III-mediated BSEP sorting preceded Rab11A-regulated apical cycling of BSEP.

**Conclusions:**

Our results showed the first example that ESCRT-III is essential for canalicular trafficking of apical membrane proteins, and provide new targets for therapeutic approaches in BSEP associated cholestasis.

## Background

The bile salt export pump (BSEP), encoded by the gene *ABCB11*, mediates the rate-limiting step of bile salt transport from hepatocytes into bile canaliculi [1]. Abnormal expression or functional disturbance of BSEP results in a spectrum of cholestatic diseases including progressive familial intrahepatic cholestasis type 2 (PFIC2), benign recurrent intrahepatic cholestasis type 2 (BRIC2), drug-induced liver injury, and intrahepatic cholestasis of pregnancy [2–4]. PFIC2 and BRIC2 are characterized by early-onset liver diseases in infancy or childhood, but the treatment options for PFIC2 and BRIC2 are very limited [5]. Without liver transplantation, PFIC2 is a fatal disease, and BRIC2 may also progress to liver cirrhosis. In addition, neonates and infants, especially the premature infants, are susceptible to cholestasis caused by immature BSEP expression or secondary insults such as infections, toxins or ischemic injuries, resulting in the disruptive localization and function of BSEP [6]. There is a need for better understanding the mechanism of BSEP-related diseases and developing potential therapeutic strategies.

**Fig. 1:**
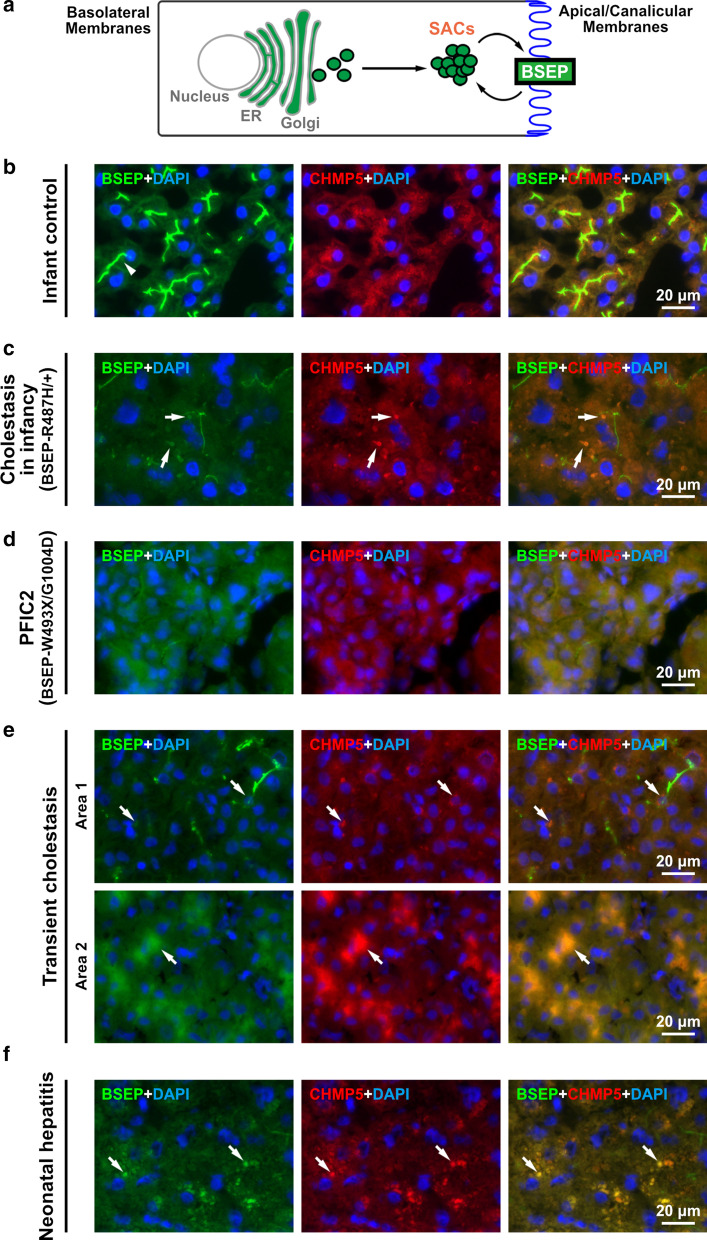
Aberrant subapical BSEP compartments in cholestatic human livers are CHMP5 positive. **a** An illustration of the post-Golgi trafficking of BSEP in hepatocytes. De novo synthesized BSEP (green) targets the subapical compartments (SACs) from the *trans*-Golgi and constitutively cycles between SACs and the apical/canalicular membrane (blue) of hepatocytes. **b**–**f** Aberrant subapical BSEP compartments in cholestatic human livers are associated with CHMP5 co-expression. Immunofluorescence staining demonstrated BSEP and the ESCRT-III CHMP5 in the subcellular compartments of hepatocytes in human liver samples from **b** a representative infant control; **c** a patient with cholestasis in infancy harboring heterozygous BSEP-R487H mutation (BSEP-R487H/ +); **d** a PFIC2 infant with BSEP-W493X/G1004D mutations; **e** a diseased child with transient cholestasis; and **f** a representative patient with neonatal hepatitis. Cryosection of human livers samples were stained for BSEP (green) and CHMP5 (red). The liver nuclei were stained with DAPI. The arrowhead indicates the apical/canalicular membrane of hepatocytes; arrows indicate the aberrant BSEP vesicles in the cytoplasm of cholestatic livers. Notably, two different patterns of BSEP and CHMP5 were observed in the sample of transient cholestasis **e** and referred to Additional file 1**: **Figure S1

BSEP is an apical/canalicular transporter with 12 transmembrane segments. Targeting to the canalicular membrane and sustaining the membrane abundancy are critical for BSEP functions. Newly synthesized canalicular proteins go through two different routes to the canalicular membrane of hepatocytes [7]. Some proteins, such as dipeptidyl peptidase-IV, traffic via the indirect targeting, in which proteins are transferred initially to the basolateral membrane and then undergo transcytosis to the apical membrane. Others, including BSEP and multidrug resistance protein 1, target to the canalicular membrane without transcytosis [8, 9]. Before sorting to the canalicular membrane, BSEP first targets to the Rab11-positive intermediate pool/subapical compartment (SAC), and then constitutively cycles between SACs and the canalicular membrane [9, 10] (Fig. 1A). This two-stepped direct targeting of BSEP suggests a complicated regulation at SACs.

We have reported the subcellular distribution of BSEP in human fetal hepatoblasts is different from that in adult hepatocytes [6]. The partially intracellular and partially canalicular distribution of BSEP in fetal livers suggested some regulations during BSEP trafficking. Besides, increased intracellular retention of BSEP is the main character of BSEP associated cholestasis [11]. The BSEP apical targeting may use some common mechanisms shared between developmental livers and cholestatic liver diseases. The mechanisms of disturbed BSEP targeting and functions that cause cholestasis have been elucidated for only a small proportion of BSEP mutational defects [12–16]. Hence, we aimed to investigate the apical targeting of BSEP and pathogenic BSEP mutations found in human cholestasis patients. In this study, we discovered that charged multivesicular body protein 5 (CHMP5), a key endosomal protein complex required for transport subcomplex-III (ESCRT-III), and its associated ESCRT machinery are essential for the polarized sorting of BSEP. ESCRTs functioning on BSEP sorting is upstream of Rab11. Aberrant association between CHMP5 and BSEP mutants may cause the intracellular retention of BSEP mutants occurred in PFIC2.

## Methods

### Human liver samples

Liver samples were obtained from three adult living-related liver donors, four infants with non-cholestatic metabolic diseases, and fetal liver samples at gestational ages of 13 and 22 weeks from elective terminations of pregnancy for medical indications under informed consent. Liver samples from 5 patients with cholestatic liver diseases were analyzed, including one 4-year-old boy with transient cholestasis, three infants with neonatal cholestasis and subsequent recovery, and one infant with severe cholestasis with a heterozygous c.1460G > A (p.R487H) BSEP mutation [12]. Informed consent was obtained from each subject, and the protocol was approved by the Institutional Review Board, National Taiwan University.

### Hydrodynamic injection of mice

Mice with targeted inactivation of the *Bsep* (*spgp*) gene on an FVB/NJ genetic background [17], and wild-type FVB/NJ mice were used for hydrodynamic injection study. Totally twenty microgram of plasmid DNA in PBS was injected hydrodynamically through mouse tail veins. Mice were sacrificed in indicated experimental times. Sera and liver samples were collected for biochemical and immunolabeling assays. Experiments were conducted according to the approved protocols from the Committee on Animal Care, National Taiwan University College of Medicine.

### Temperature shift assay

The temperature shift assay was modified from the protocol described previously [18]. Cells were incubated at 40 °C, 30 °C, and 37 °C sequentially to synchronize proteins at the ER, Golgi’s apparatus, and to release into the post-Golgi network, respectively. The protocol is indicated in each figure.

### Yeast two-hybrid screen and interaction assay

The yeast two-hybrid screen and interaction assay were performed using the “interaction-trap” system [19]. The human fetal liver cDNA library (Clontech Laboratories, Mountain View, CA) was screened using amino acid residues 491-680 of human BSEP as bait. Genes encoding different BSEP protein fragments for interacting-domain mapping were cloned and subjected to the same assay system of yeast two-hybrid.

### Plasmid construction and transfection

Human BSEP-expressing constructs were cloned from pTRE-tight-SPGP, a kindly gift of Dr. Victor Ling (The University of British Columbia, Vancouver, Canada). The cryptic prokaryotic promoter in human BSEP coding sequence [20] was first inactivated via site-directed mutagenesis and then sub-cloned into Hind III/Kpn I site of p3XFLAG-CMV-10 and pEGFP-C2 to yield p3XFLAG-BSEP and pEGFP-BSEP, respectively. A synthetic poly-nucleotide encoding a (GGGGS)_3_ polypeptide linker was further inserted in Bgl II/Hind III of pEGFP-BSEP to generate pEGFP-LK-BSEP. All the other plasmids expressing full-length or fragmental BSEP and BSEP mutants were cloned from pEGFP-LK-BSEP.

The open-reading frame (ORF) of *CHMP5* (NM_016410.5), *VPS4A* (NM_013245.2), *VPS4B* (NM_004869.4), and *Rab11A* (NM_004663) was cloned from the cDNA of Hep G2 cells into pCMV6-AC-3HA, pmCherry-C1 or pEGFP-C1 to obtain pCHMP5-3HA, pmCherry-CHMP5, pmCherry-VPS4A, pmCherry-VPS4B, and pEGFP-Rab11A. Site-directed mutagenesis was further used to generate plasmids expressing the dominant-negative mutants of VPS4A-E228Q (VPS4A-EQ), VPS4B-E235Q (VPS4B-EQ), or Rab11A-S25N (Rab11A-SN). A (GGGGS)_3_-coding linker was inserted into Bgl II/Hind III of pmCherry-CHMP5 to generate pmCherry-LK-CHMP5.

The plasmid pcDNA3.1 ( +)-Mem-DsRed-Monomer was obtained from the Biomedical Resource Core and the Imaging Core at the First Core Lab, National Taiwan University College of Medicine. Plasmids expressing HA-tagged ubiquitin (HA-Ub) mutants were obtained from Addgene, including pRK5-HA-Ubiquitin-WT (#17608), -K48 (#17605), -K48R (#17604), and -K63 (#17606) [21]. The plasmid pRK5-HA-Ub-K63R was constructed using site-directed mutagenesis from pRK5-HA-Ubiquitin-WT. Plasmids expressing short hairpin RNA (shRNA) targeting mouse *Chmp5* (TRCN0000009719 and TRCN0000009721) and the paired scramble control (pLAS-Void) were purchased from National RANi Core Facility Platform, Academia Sinica, Taiwan. The target sequences for mouse *Chmp5* were as follows (coding strand sequence indicated): TRCN0000009719, 5′-CCAACCAGATTTAGGTTTCTT-3′ and TRCN0000009721, 5′-CCTGCTAAGAACATGGTCAAA-3′. The shRNA scramble sequence of pLAS-Void was 5′-AGTTCAGTTACGATATCATGTCTCGAGACATTCGCGA GTAACTGAACTTTTTTG-3′. The deliveries of plasmid DNA and small interference RNA (siRNA) were performed using Lipofectamine™ 3000 and Lipofectamine™ RNAiMAX (Thermo Fisher Scientific, Waltham, MA), respectively.

### Cell culture

Human hepatoma cell lines Hep G2 [HEPG2] (ATCC® HB-8065™), Huh-7, and Mahlavu were cultured in DMEM with high glucose supplement with 10% fetal bovine serum, MEM-nonessential amino acids, sodium pyruvate, GlutaMAX™, and antibiotics. All cell-culture related reagents were purchased from Thermo Fisher Scientific.

### Protein extraction and subcellular fractionation

For the extraction of total cell lysates, cultured cells were washed using cold PBS and lysed using denaturing lysis buffer (150 mM NaCl; 50 mM Tris–Cl, pH 8.0; 1% NP-40; 0.5% sodium deoxycholate; 0.1% SDS; 5 mM EDTA) or non-denaturing n-dodecyl β-d-maltoside (β-DDM) lysis buffer (150 mM NaCl; 20 mM HEPES, pH 7.4; 0.1% β-DDM; and 1 mM EDTA) supplement with protease and phosphatase inhibitor cocktail (Thermo Fisher Scientific). To collect the supernatant, lysed cells were centrifuged at 4 °C with 13,000×*g* for 10 min. For isolating total membrane proteins, cells were fractionated using the Mem-PER™ Plus Membrane Protein Extraction Kit (Thermo Fisher Scientific). Plasma-membrane and organelle-membrane proteins were isolated by Trident Membrane Protein Extraction Kit (GeneTex; Taiwan). All ubiquitination-related experiments were further supplement with 20 mM N-ethylmaleimide (NEM) in lysis buffers or reagents.

### Immunoprecipitation and Western Blotting

Immunoprecipitation was performed using Dynabeads Protein G (Thermo Fisher Scientific) conjugated with antibodies or normal immunoglobulins. GFP-Trap (ChromoTek GmbH, Germany) and anti-HA magnetic beads (Thermo Fisher Scientific) were used to immunocapture EGFP-tagged and HA-tagged proteins, respectively. All protein samples were eluted in reducing Bolt™ LDS Sample Buffer; separated on 4–12% Bolt™ gels (Thermo Fisher Scientific), and electro-transferred to PVDF membranes for immunoblotting of targeting proteins. The signal density was quantified using Image Lab (Bio-Rad, Hercules, CA).

### Immunostaining

Cryopreserved human and mouse liver tissues were sectioned in 6 μm and fixed in cold acetone. For in vitro cell samples, cells were fixed and permeabilized using 4% paraformaldehyde and 0.5% Triton X-100. After serum blocking, samples were incubated with primary antibodies and visualized by fluorochrome-conjugated secondary antibodies. Samples were counter-stained and mounted using ProLong Diamond Antifade Mountant with DAPI (Thermo Fisher Scientific).

For immunohistochemical staining, paraffin-embedded mouse liver samples were sectioned in 4 μm and then subjected to de-wax, rehydrate, and antigen retrieval. Endogenous peroxidase activity and biotins were quenched using hydrogen peroxide and Avidin/Biotin Blocking Kit (SP-2001, Vector Laboratories), respectively. After incubation of primary antibodies and biotinylated secondary antibodies, tissue samples were visualized using VECTASTAIN Elite ABC HRP Kit (PK-6100; Vector Laboratories) and VECTOR NovaRed Peroxidase Substrate Kit (SK-4800, Vector Laboratories). Cell nuclei were stained using hematoxylin.

### RNA interference and quantitative RT-PCR

Pre-designed small interference RNA (siRNA) pool targeting human *CHMP5* (si-CHMP5; L-004697-01) and the paired non-targeting control (si-CTL; D-001810-10) were purchased from Dharmacon (Lafayette, CO). Hep G2 cells on 6-well plate were reverse transfected with 10 nM siRNA (25 pmol per well). The mRNA and protein of treated cells were harvested to evaluate knockdown efficiency via quantitative PCR and immunoblotting, respectively. Total RNA was extracted through TRIzol^®^ reagent (Thermo Fisher Scientific). Two micrograms of total RNA were reverse transcribed using SuperScript III^®^ (Thermo Fisher Scientific) with oligo-dT primers, and then subjected to duplex quantitative PCR using TaqMan™ Gene Expression Assays (Thermo Fisher Scientific) for *CHMP5* (Hs00995158_g1) and *GAPDH* (Hs02758991_g1).

### Determination of total bile acids

Hep G2 cells were pre-treated with siRNA for 48 h, and then transfected with pEGFP-LK-BSEP plus pmCherry-LK-CHMP5 or its vector pmCherry-C1 48 h. Conditional medium was collected for determining total bile acids using Total Bile Acids Assay Kit (DZ042A; Diazyme Laboratories, Poway, CA).

### Image acquisition and processing

Immunostaining samples were imaged using ZEISS Observer. D1 fluorescence microscope (Carl ZEISS, Jena, Germany) equipped with EC Plan-Neofluar 10x/0.30 M27, LD Plan-Neofluar 20x/0.4 Korr Ph2 M27, and LD Plan-Neofluar 40x/0.6 Korr Ph2 M27 objectives; DAPI, FITC, and TRITC filters. AxioCam mRM r3.1 CCD camera and the microscope software AxioVision were used to image acquisition. For confocal microscopy, ZEISS LSM 880 with the Airyscan mode and ZEN software were used. All images were processed by Adobe Photoshop CS6 or Affinity Photo.

### Subcellular distribution index and canalicular targeting index

Subcellular distribution index was calculated from images obtained from examined Hep G2 cells after temperature shift assay and immunostaining of FLAG-BSEP proteins and Mem-DsRed. The numbers of cells with both FLAG-BSEP and Mem-DsRed signals (FLAG-BSEP + Mem-DsRed) and, from these double-positive cells, with FLAG-BSEP signals at the plasma membrane (pFLAG-BSEP) were counted and then calculated as followed:

$$\mathrm{Subcellular distribution index }\left(\mathrm{\%}\right)= \frac{\mathrm{No}.\mathrm{ of cells with pFLAG}-\mathrm{BSEP}}{\mathrm{No}.\mathrm{ of cells with FLAG}-\mathrm{BSEP}+\mathrm{Mem}-\mathrm{DsRed}}$$

Canalicular targeting index was calculated from images obtained from hydrodynamically injected mouse liver samples with FLAG-BSEP staining. The number of canalicular FLAG-BSEP (cFLAG-BSEP) was manually calculated, and the area of the mouse live tissue in each image was determined by ImageJ program. The canalicular targeting index was calculated as followed:

$$\mathrm{Canalicular targeting index}= \frac{\mathrm{No}.\mathrm{ of cells with cFLAG}-\mathrm{BSEP}}{\mathrm{Area of the mouse liver tissue in images}}$$

### Statistical analysis

All experiments are represented as mean ± SD of independently triplicated or quadruplicated experiments. Statistical significance was determined using two-tailed unpaired Student’s *t*-test, and *p* values ≤ 0.05 were consider significant (*P ≤ 0.05). Data were analyzed using GraphPad Prism (GraphPad Software, San Diego, CA).

## Results

### Cholestatic human livers demonstrate aberrant subapical BSEP vesicles

To study the subcellular distribution of BSEP in cholestatic human hepatocytes, two cholestatic human liver samples with BSEP mutations were analyzed via immunofluorescence assays. One liver sample is from a severely cholestatic infant harboring a heterozygous BSEP-R487H mutation (BSEP-R487H/+) and the other is a PFIC2 infant with compound heterozygous BSEP mutations (BSEP-W493X/G1004D). In comparison with the age-matched infant control liver samples, the level of canalicular BSEP was reduced in both cholestatic livers, a character in BSEP associated cholestasis. On the other hand, abnormally enlarged or scattered puncta with BSEP-positive signals were formed in the subapical area in the cholestatic hepatocytes, which were different from that in the control liver (Fig. 1b–d; the left panel). To confirm the findings of aberrant subapical BSEP distribution in cholestatic hepatocytes, we further analyzed one transient cholestasis and three neonatal hepatitis liver samples. Similarly, huge cytoplasmic compartments or aggregated vesicles with BSEP-positive signals were observed in the transient cholestasis (the left and the top panels of Fig. 1e and Additional file 1: Figure S1, respectively) and in these neonatal hepatitis (Fig. 1f; the left panel) liver samples, respectively. The findings of BSEP localizing in aberrant SACs suggest that the polarized trafficking of BSEP may be perturbed in cholestasis livers.

### The ESCRT-III CHMP5 interacts with BSEP

**Fig. 2:**
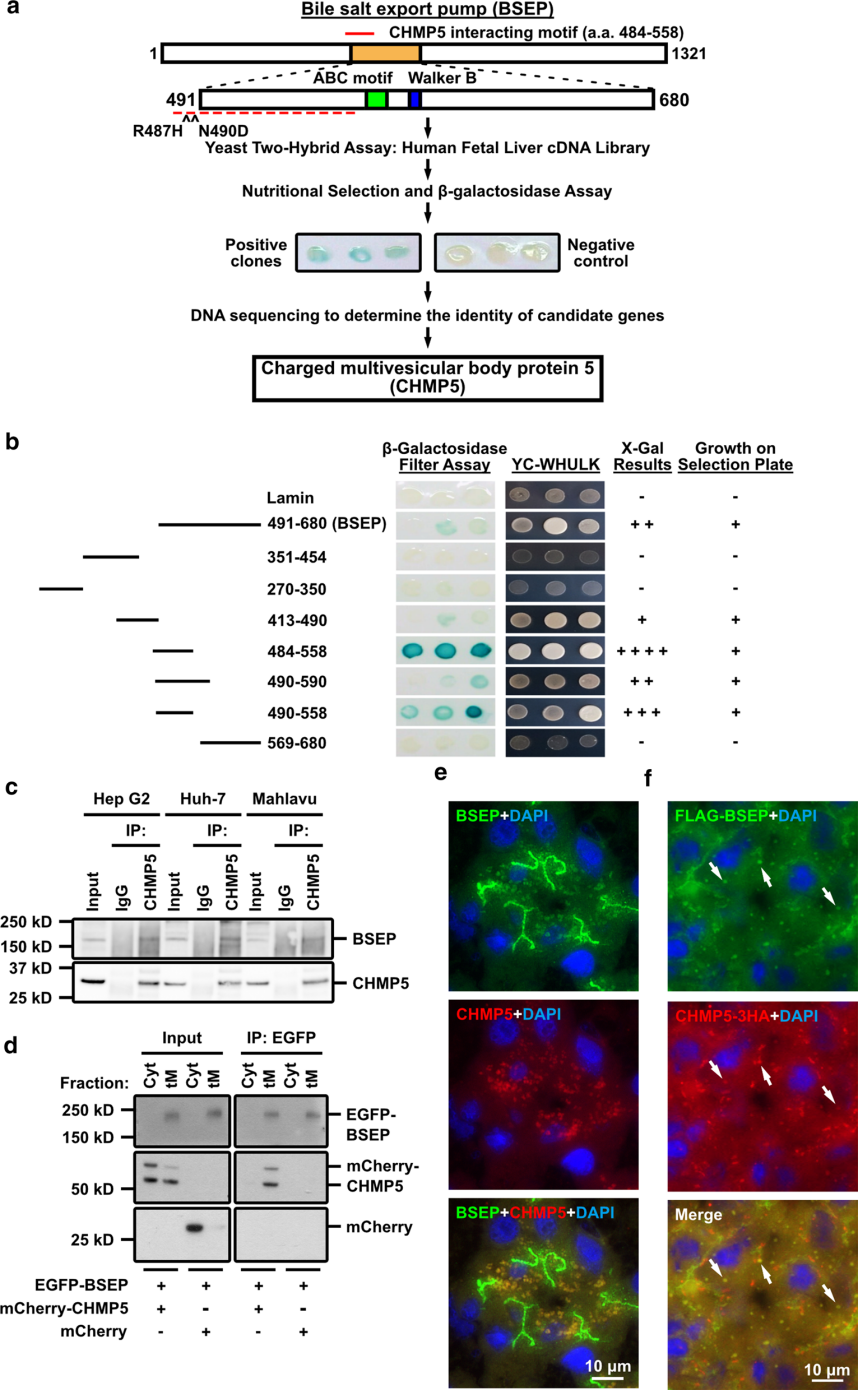
The ESCRT-III subunit CHMP5 co-localizes with BSEP in the subapical compartments of hepatocytes.** a** The flow chart illustrates the identification of CHMP5 as a BSEP-interacting protein via yeast two-hybrid screening. A positive β-galactosidase assay indicates a polypeptide (CHMP5) interacting with the BSEP bait. Lamin was used as a negative control. **b** CHMP5 interacts with the polypeptide spanning amino acid 484–558 of human BSEP. The CHMP5-interacting domain of BSEP was mapped via yeast two-hybrid screening. Various BSEP fragments were co-expressed with CHMP5 in competent yeast cells and screened by nutritional depletion and β-galactosidase filter assays. **c** BSEP was co-immunoprecipitated with CHMP5 from three human hepatoma cell lines, Hep G2, Huh-7, and Mahlavu. Total cell lysate (25 μg) was loaded as an input control; normal mouse immunoglobulins (IgG) were used as a negative control of immunoprecipitation. **d** Endogenous and mCherry-tagged CHMP5 were co-immunoprecipitated with EGFP-BSEP in the total membrane-protein fraction. Hep G2 cells co-expressing EGFP-BSEP and mCherry-CHMP5 or mCherry were fractionated into cytosolic protein (Cyt) and total membrane-protein (tM) fractions. The tM fraction contains the transmembrane and membrane-associated proteins (referred to Additional file 1: Figure S2). The two fractions were subjected to immunoprecipitation and immunoblotting via the antibodies indicated. **e** Immunofluorescence staining demonstrates that CHMP5 co-localizes with subapical BSEP in the adult human livers. Cryosection of adult human liver samples was stained for BSEP (green) and CHMP5 (red). The liver cell nuclei were stained with DAPI. **f** Immunofluorescence assays reveal the co-localization of CHMP5-3HA and FLAG-BSEP in the cytoplasmic vesicles in *Bsep* knockout mouse livers. CHMP5-3HA and FLAG-BSEP were co-expressed in *Bsep* knockout mice via hydrodynamic injection. Cryosections of the mouse liver samples were stained by anti-FLAG and anti-HA antibodies for FLAG-BSEP (green) and CHMP5-3HA (red), respectively. The liver cell nuclei were stained with DAPI. Arrows indicate the subapical vesicles with both FLAG-BSEP and CHMP5-3HA signals

We hypothesized that the BSEP retention in the SACs of these cholestatic hepatocytes is likely caused by apical trafficking defects. To address the hypothesis, we first identified cellular BSEP-binding partners involving in membrane trafficking by using the human BSEP amino acids (a.a.) 491–630, within which plenty of BSEP mutations have been reported [4], as bait to screen a human fetal liver cDNA library via yeast two-hybrid system. Charged multivesicular body protein 5 (CHMP5), a subunit of the endosomal protein complex required for transport subcomplex-III (ESCRT-III), was found to be one of the BSEP interacting candidates (Fig. 2a). We picked CHMP5 among the candidate proteins with moderate interaction for its potential physiological role in BSEP trafficking and localization (Additional file 2: Table S1). By mapping with different fragments of BSEP via the yeast two-hybrid assay, the CHMP5 interacting domain of BSEP mainly located within a.a. 484-558, which is harbored in the first nucleotide-binding fold (NBF1; a.a. 414-610) of human BSEP (Fig. 2b).

The interaction between BSEP and CHMP5 was validated via co-immunoprecipitation using three hepatoma cell lines, Hep G2, Huh-7, and Mahlavu. As shown in Fig. 2c, endogenous BSEP was co-immunoprecipitated with CHMP5 in these cell lines. We have depleted the cellular BSEP using an siRNA poll targeting human *ABCB11/BSEP* to ascertain the endogenous signal of BSEP (data not shown). BSEP is a transmembrane protein, whereas CHMP5 is a cytoplasmic protein distributed as either cytosolic monomers or cytoplasmic vesicle-membrane associated polymers. Total membrane-protein fractionation was used to study the subcellular interaction site of BSEP and CHMP5. After total membrane-protein fractionation, BSEP was found to distribute in the total membrane-protein fraction (tM), which consists of plasma membrane (PM) and organelle-membrane (OM) protein fractions (Fig. 2d and Additional file 1: Figure S2). Consistent with previous studies, endogenous and mCherry-tagged CHMP5 was predominantly expressed in the cytosol (Cyt), with less in the tM fractions (Fig. 2d and Additional file 1: Figure S2). Immunoprecipitation using anti-EGFP antibodies revealed endogenous and mCherry-tagged CHMP5 formed complexes with EGFP-BSEP within the tM fraction (Fig. 2d). These data suggest that only membrane-associated CHMP5 interacts with BSEP.

### Subapical BSEP localizes in CHMP5-positive subapical compartments

To study the interaction between BSEP and CHMP5 in vivo, adult human liver samples were analyzed and a hydrodynamic injection-based mouse model was established [22]. Immunofluorescence assay of BSEP and CHMP5 in adult human liver sections revealed that CHMP5 co-localized with subapical BSEP (Fig. 2e and Additional file 1: Figure S3). In addition to CHMP5, these subapical BSEP vesicles were also co-localized with the CHMP5 interacting ESCRT protein LIP5 (Additional file 1: Figure S3). We applied hydrodynamic injection to co-express FLAG-BSEP and haemagglutinin (HA)-tagged CHMP5 (CHMP5-3HA) in *Bsep* knockout mice livers [17]. The liver sections of these mice were analyzed through immunofluorescence assays. In line with the total membrane-protein fractionation results in vitro and the observation in adult human liver sections (Fig. 2d, e), the CHMP5-3HA signal displayed a mainly cytoplasmic distribution as speckles co-localizing with subapical BSEP (Fig. 2f). Therefore, the SACs of hepatocytes, in which the subapical BSEP localizes, are CHMP5 positive.

### The canalicular targeting of BSEP is developmentally regulated and associated with CHMP5

We discovered the SACs being CHMP5 positive, which prompted us to examine the abnormal subapical BSEP puncta observed in the above-mentioned cholestatic liver samples on CHMP5 expression. In these cholestatic hepatocytes, the aberrant subapical BSEP puncta were all CHMP5 positive (Fig. 1). These observations on cholestatic liver samples raised a question: whether the different patterns of BSEP distribution in fetal and adult livers [6] may associate with different CHMP5 distribution. The expression and distribution of BSEP and CHMP5 in the human liver samples of fetus at gestational age 13–22 weeks, infant, and adult were analyzed. As shown in Additional file 1: Figure S4, the canalicular BSEP signals were gradually increased during hepatocyte maturation; the cytoplasmic/subapical BSEP gradually formed clear puncta at the SACs adjacent to the canalicular membrane of infant and adult human hepatocytes. The CHMP5 signals revealed remarkable association to the cytoplasmic/subapical BSEP signals. Moreover, some CHMP5 single-positive vesicles aligned with the canalicular BSEP were observed in fetal hepatoblasts, especially in the fetal livers at gestational age 18–22 weeks (Additional file 1: Figure S4). Hence, these results suggested that the apical sorting of BSEP is developmentally regulated, which is also very likely associated with the ESCRT-III CHMP5. The defective apical expression of BSEP in cholestatic hepatocytes may be caused by impaired CHMP5-associated ESCRT functions.

### Aberrant interaction between BSEP mutants and CHMP5 affects the polarized trafficking of BSEP mutants

The ESCRT complex participates in the endocytic pathway [23]; however, whether ESCRTs involving the apical membrane trafficking is unknown. To dissect the interaction between human BSEP mutants and CHMP5, we constructed plasmids expressing FLAG-tagged BSEP-R487H or BSEP-N490D, another PFIC2-causing BSEP mutant but its apical expression partially retained [13], for in vitro and in vivo studies.

**Fig. 3:**
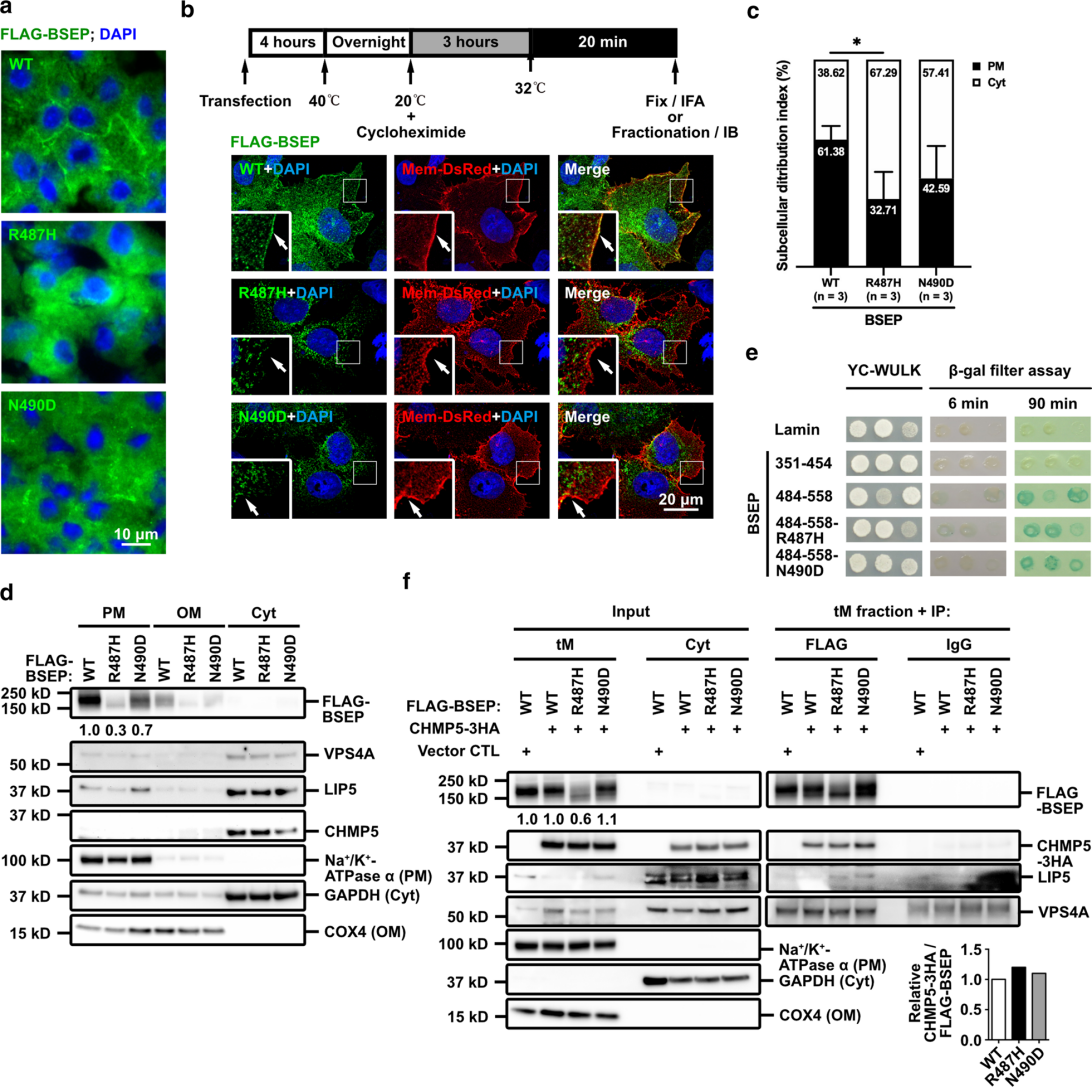
Aberrant interaction with CHMP5 affected the polarized trafficking of BSEP-R487H and BSEP-N490D mutants.** a** Representative immunostaining demonstrates the impaired canalicular targeting of BSEP-R487H and BSEP-N490D mutants at the steady state in vivo. FLAG-BSEP wild type and mutants were expressed in *Bsep* knockout mouse livers for 7 days via hydrodynamic injection. **b** Confocal images reveal the impaired post-Golgi trafficking and membrane targeting of BSEP-R487H and BSEP-N490D mutants. Temperature shift assay was performed with Hep G2 cells co-expressing the membrane marker Mem-DsRed plus FLAG-tagged BSEPs (stained with anti-mCherry and anti-FLAG antibodies, respectively). Arrows indicate the plasma membrane. **c** The bar graph (n = 3, mean ± SD) illustrates the subcellular distribution index of FLAG-BSEP wild-type and the two mutations after temperature shift assays. Total 98 to 131 cells were counted. (*P ≤ 0.05). **d** Immunoblotting reveals the decreased plasma-membrane targeting of BSEP-R487H and BSEP-N490D. Hep G2 cells expressing FLAG-BSEP wild-type or two mutants were subjected to temperature shift assay followed by subcellular fractionation. The plasma-membrane (PM), organelle-membrane (OM) and cytosolic (Cyt) fractions were analyzed to detect the indicated proteins and the fractionation control. Numbers are the relative ratio of the signal density of FLAG-BSEPs in the PM normalized to that of the PM control. **e** BSEP polypeptides (amino acids [a.a.] 484-558) harboring either R487H or N490D mutations could interact with CHMP5 in yeast two-hybrid assays. The BSEP-484-55 and BSEP-351-454 fragments were used as the positive and negative CHMP5-interaction control, respectively. Lamin was negative control. **f** Immunoblotting demonstrates aberrant association between the two BSEP mutants and ESCRT-III molecules. Hep G2 cells were co-expressed CHMP5-3HA and FLAG-BSEP-WT or the BSEP mutants and then fractionated. The pCMV6-AC-3HA was the vector control (Vector CTL). FLAG-tagged BSEP proteins in the total membrane (tM) fractions were immunoprecipitated and probed the indicated proteins. The bar graph illustrates the relative signal density normalized to FLAG-BSEPs of the “tM fraction + IP” panel

We first tested whether both BSEP mutants at the steady state indeed had disturbances in canalicular targeting in vivo by using hydrodynamic injection-mediated mouse models. Immunofluorescence assay of the liver samples from hydrodynamically injected mice displayed decreased canalicular FLAG signals of FLAG-BSEP-R487H and FLAG-BSEP-N490D in comparison with wild-type FLAG-BSEP. Moreover, strong cytoplasmic signals were observed in mouse hepatocytes with p3XFLAG-BSEP-R487H injection (Fig. 3a). Temperature shift assays to probe post-Golgi trafficking of plasma membrane proteins, combined with immunostaining or plasma membrane protein fractionation, were performed with Hep G2 cells expressing FLAG-BSEP and two FLAG-BSEP mutants [18]. Mem-DsRed was served as a cellular membrane marker for cell image analysis. As shown in Fig. 3b, c, both BSEP mutants, especially BSEP-R487H, revealed defective post-Golgi membrane targeting in Hep G2 cells. Similarly, immunoblotting demonstrated decreased plasma membrane targeting of the two BSEP mutants in fractionated cell samples (Fig. 3d). Taken together, these results suggest the apical targeting of both BSEP-R487H and BSEP-N490D mutants is indeed impaired in different degrees and in line with the observation in Fig. 1c and in the literature [13].

We hypothesized that abnormal association with ESCRT molecules caused the impaired apical sorting of both BSEP mutants. The interaction between both mutants and CHMP5 was analyzed through yeast two-hybrid assay and co-immunoprecipitation. CHMP5 together with either BSEP (484-558)-R487H or BSEP (484-558)-N490D was co-expressed in yeast cells and subjected to nutritional selection as well as β-gal filter assays. BSEP (484-558) and BSEP (351-454) was used as positive and negative CHMP5-interaction control, respectively. As shown in Fig. 3e, BSEP (484-558) harboring either R487H or N490D mutations could interact with CHMP5.

Total membrane-protein fractionation and co-immunoprecipitation were used to dissect the interaction between CHMP5 and the two BSEP mutants. Immunoblotting of the fractionated samples revealed the decreased expression of BSEP-R487H mutant (Fig. 3f, the input control panel). Immunoprecipitation via anti-FLAG antibodies was performed with the tM fractions of the cells co-expressing CHMP5-3HA and either FLAG-BSEP mutant constructs. In accordance with the result of Fig. 3e, either FLAG-BSEP-R487H, FLAG-BSEP-N490D or FLAG-BSEP wild type formed complexes with CHMP5-3HA and two ESCRT molecules LIP5 and VPS4. However, there was a trend that more CHMP5-3HA was co-captured with FLAG-BSEP-R487H (Fig. 3f, the right panel and the bar graph). Hence, the defective apical targeting of BSEP-R487H and BSEP-N490D may be caused by altered affinity between the mutated BSEP and the ESCRT-III CHMP5.

### CHMP5 regulates the apical targeting of BSEP and BSEP-mediated bile acid secretion

BSEP-R487H and BSEP-N490D revealed an increase in CHMP5 association but a decrease in apical sorting. We hypothesized that CHMP5 and the ESCRT machinery may participate in the post-Golgi and apical trafficking of BSEP. To explore whether CHMP5 indeed affects the apical trafficking of BSEP, small interference RNA (siRNA) and short hairpin RNA (shRNA) targeting to *CHMP5/Chmp5* mRNA were used in Hep G2 cells and in a hydrodynamic injection-based mouse model, respectively. Both the mRNA and protein levels of CHMP5 can be knocked down and sustained at steady state from 48 to 96 h post-transfection (Additional file 1: Figure S5a, b). Hence, further studies using exogenously expressed BSEP with *CHMP5* knockdown were performed 48 h after 10-nM siRNA delivery. Besides, the protein level of endogenous BSEP was unaffected by *CHMP5* knockdown (Additional file 1: Figure S5C–S5E), which indicates that CHMP5 does not affect the protein expression or turnover of BSEP.

**Fig. 4:**
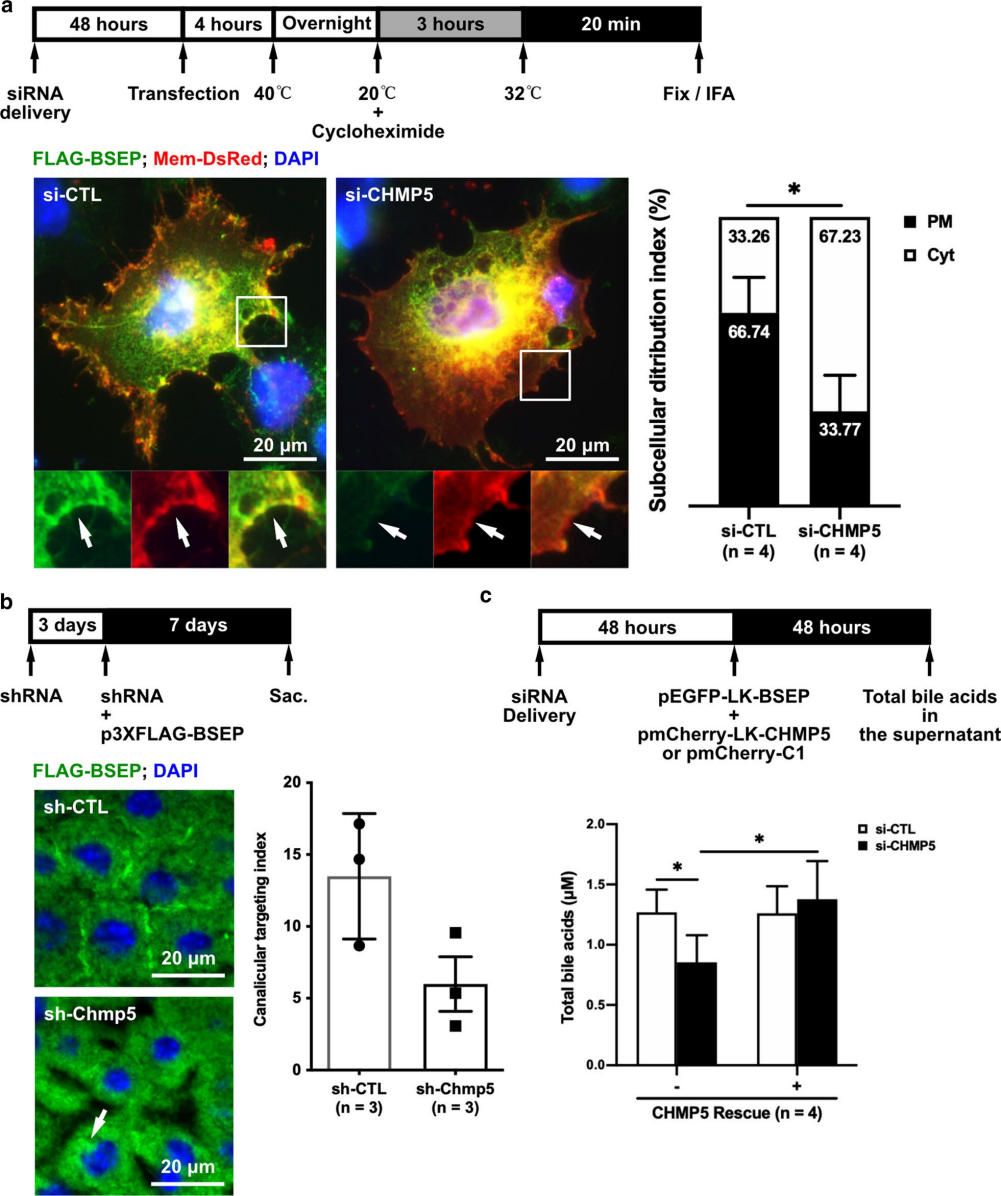
CHMP5 regulates the apical targeting of BSEP and BSEP-mediated bile acid secretion.** a** Representative images demonstrate the impaired membrane targeting of BSEP in CHMP5-knockdown Hep G2 cells. Hep G2 cells were pretreated with *CHMP5*-targeting (si-CHMP5) or non-targeting (si-CTL) siRNA pools, and then co-expressed FLAG-BSEP and the membrane marker Mem-DsRed for temperature shift assays. Cell nuclei, FLAG-BSEP and Mem-DsRed were stained by DAPI, anti-FLAG and anti-mCherry antibodies, respectively. For clarity, the squared images are enlarged versions of the white open squares; arrows indicate the FLAG-BSEP on the plasma membrane. The bar graph (n = 4, mean ± SD) illustrates the subcellular distribution index, in which total 121 and 96 cells in the si-CTL and the si-CHMP5 groups, respectively, were analyzed. Two-tailed unpaired *Student’s t*-test was used (*P = 0.0286). **b** Representative images reveal defective apical targeting of BSEP in *Chmp5*-knockdown mouse livers. The plasmid pool expressing shRNA targeting mouse *Chmp5* (sh-Chmp5) or the scramble sequence (sh-CTL) and p3XFLAG-BSEP were hydrodynamically injected into FVB/NJ mouse livers. The distribution of FLAG-BSEP (green) in these liver samples were immunostained by anti-FLAG antibodies. The arrow indicates the accumulated subapical BSEP. The bar graph (n = 3, mean ± SD) illustrates the canalicular distribution of FLAG- BSEP in *Chmp5*-knockdown mouse livers. More than 10 images per mouse and three mice in each group were analyzed for the canalicular targeting index. **c** The bar graph (n = 4; mean ± SD) illustrates that BSEP-mediated bile acid secretion was reduced in *CHMP5*-knockdown cells and rescued through overexpressing CHMP5. The total bile acids concentrations were determined from the conditional medium of Hep G2 cells with indicated transfection. The plasmid pmCherry-C1 was used as a negative control for CHMP5 rescue. This experiment was performed in quadruplicate. Two-tailed unpaired *Student’s t*-test was used (*P ≤ 0.05). The *P*-value is 0.0314 and 0.0402 of the left and the right analysis, respectively

Temperature shift assays were performed with cells expressing FLAG-BSEP for evaluating its post-Golgi targeting in CHMP5 depletion. Hep G2 cells, pre-treated with 10 nM of si-CHMP5 or si-CTL, were co-expressed with FLAG-BSEP and Mem-DsRed. In CHMP5 depleted cells, plasma membrane targeting of FLAG-BSEP was significantly decreased (*P* = 0.0286) and FLAG-BSEP was accumulated in the cytoplasm, especially the perinuclear region (Fig. 4a). CHMP5-mediated BSEP apical-targeting was further examined in vivo via the hydrodynamic injection-mediated mouse model (Fig. 4b). The plasmid pool expressing shRNA against mouse *Chmp5* (sh-Chmp5) was injected into FVB mice. In comparison with sh-CTL treated mice, a trend of diminished canalicular FLAG-BSEP and accumulated subapical FLAG-BSEP was observed in mice treated with sh-Chmp5 (Fig. 4b). The results from the *CHMP5/Chmp5*-knockdown Hep G2 cells and mouse livers showed that CHMP5 is required for the membrane targeting of BSEP in vitro and in vivo.

We next addressed the question that whether CHMP5 could impact the bile acid export function of BSEP. Compared to si-CTL pre-treated Hep G2 cells (1.269 ± 0.189 μM), the concentration of total bile acids in the conditional medium of the si-CHMP5 pre-treated cells (0.855 ± 0.225 μM, *P* = 0.0314) was significantly reduced, which could be rescued through ectopically expressed CHMP5 (1.377 ± 0.317 μM, *P* = 0.0402) (Fig. 4c). Hence, CHMP5 could affect BSEP-mediated bile acid excretion possibly through influencing the membrane targeting of BSEP.

### K63-linked ubiquitination of BSEP is required for the BSEP apical-targeting via CHMP5-associated ESCRT machinery

**Fig. 5:**
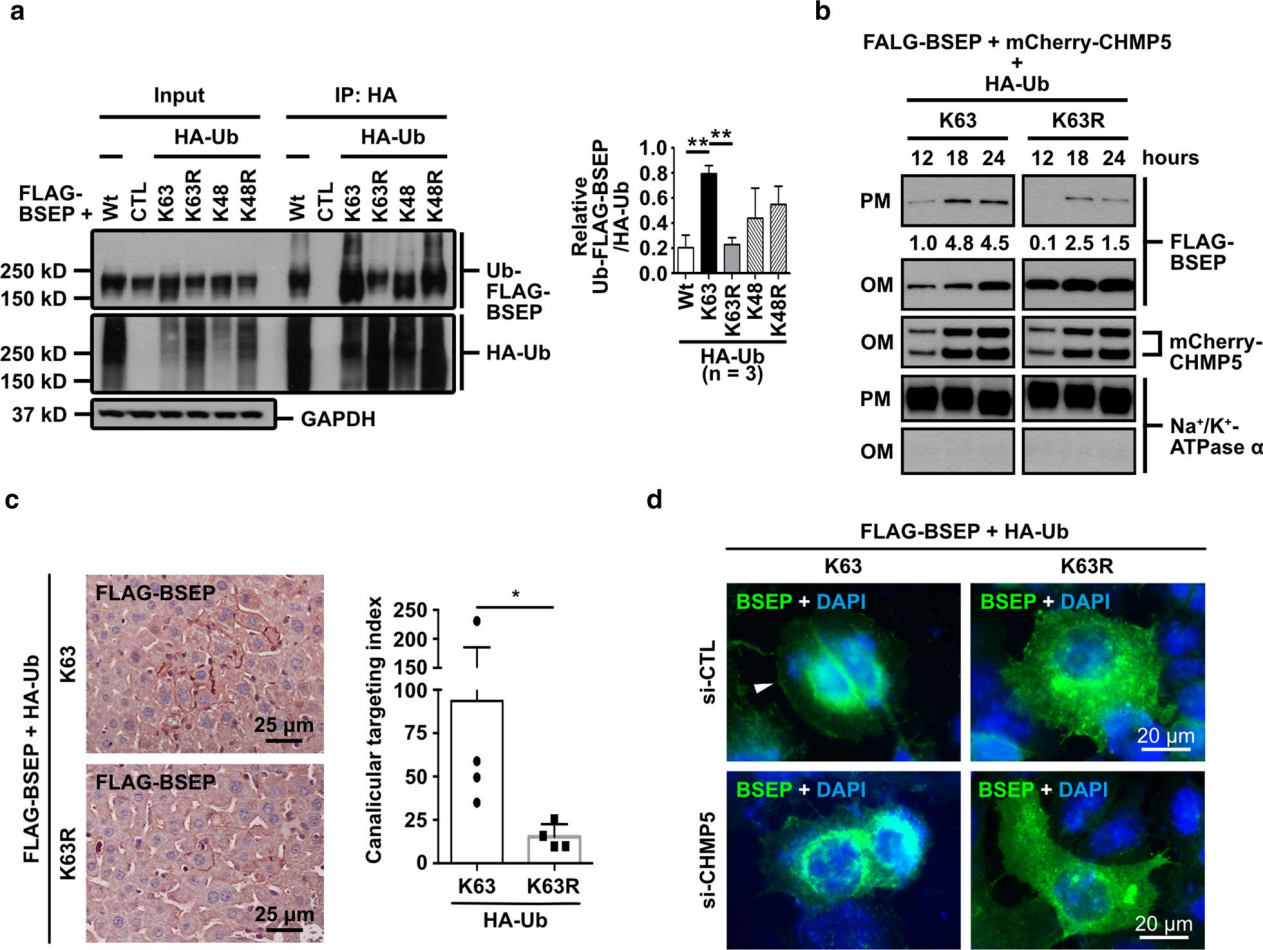
K63-linked ubiquitination of BSEP is essential for CHMP5-mediated apical targeting of BSEP.** a** BSEP was abundantly modified with K63-linked ubiquitin (Ub) chains. Hep G2 cells were co-transfected with p3XFLAG-BSEP and HA-Ub mutants or the control plasmid pcDNA3.1 ( +) for 24 h. Proteins with HA-Ub modification were isolated by anti-HA antibodies, and then detected ubiquitinated FLAG-BSEP (Ub-FLAG-BSEP) and HA-Ub linked proteins via anti-FLAG and anti-HA antibodies, respectively. The bar graph (n = 3, mean ± SD) represents the Ub-FLAG-BSEP signals normalized to HA-Ub. Two-tailed unpaired Student’s *t*-test was used (**P ≤ 0.01). **b** K63-linked ubiquitination of BSEP is important to the plasma-membrane targeting of BSEP. Hep G2 cells co-expressing FLAG-BSEP, mCherry-CHMP5 and HA-Ub-K63 or -K63R were fractionated at the indicated times. The plasma-membrane (PM) and organelle-membrane (OM) protein fractions were immunoblotted by anti-FLAG, anti-CHMP5, and anti-Na^+^/K^+^-ATPase antibodies. Na^+^/K^+^-ATPase was a PM fractionation control. Relative FLAG-BSEP signals normalized to the PM control were shown. **c** Immunohistochemical staining shows the strong canalicular FALG-BSEP in Ub-K63 co-expression in vivo. FLAG-BSEP and HA-Ub-K63 or -K63R were expressed in FVB/NJ mouse livers via hydrodynamic injection. The FLAG-BSEP was visualized by anti-FLAG antibodies and NovaRed; cell nuclei were stained by hematoxylin. The bar graph (n = 1, mean ± SD) illustrates the canalicular FLAG-BSEP in Ub-K63 or -K63R expressing mouse livers. Four images per mice and one mouse per group were analyzed for the canalicular targeting index. Two-tailed unpaired Student’s *t*-test was used (*P ≤ 0.05). The *P*-value is 0.0286. **d** Representative immunofluorescence images demonstrate Ub-K63 is essential for CHMP5-regulated membrane trafficking of BSEP in vitro. Hep G2 cells with indicated siRNA pre-treatment were co-expressed FLAG-BSEP and HA-Ub-K63 or -K63R for temperature shift assay followed by immunostainings of FLAG-BSEP (green). Cell nuclei were stained via DAPI. The arrowhead indicates the FLAG-BSEP at the plasma membrane

K63-linked ubiquitination, the covalent bonding of ubiquitin (Ub) to its targets via its K63 residue, is a specific signal for cargo sorting in the ESCRT machinery [24]. Hence, we speculated that BSEP was also ubiquitinated with K63 linkage for its ESCRT-mediated apical sorting. To approach the speculation, HA-tagged ubiquitinated proteins were immunoprecipitated from the lysate of Hep G2 cells co-expressing FLAG-BSEP and HA-tagged wild-type ubiquitin (HA-Ub-Wt) or lysine-to-arginine-substituted Ub mutants (HA-Ub-K48, -K48R, -K63, and -K63R) [21]. HA-tagged ubiquitinated FLAG-BSEP (Ub-FLAG-BSEP) greatly increased with HA-Ub-K63 co-expression, but reduced with HA-Ub-K63R co-expression. In contrast, Ub-FLAG-BSEP decreased in HA-Ub-K48 but increased in HA-Ub-K48R (Fig. 5a). These results suggest that BSEP is mainly polyubiquitinated via K63 linkage.

We further dissected the role of K63-linked ubiquitination in CHMP5-mediated membrane targeting of BSEP in vitro and in vivo. The PM fractions were isolated from Hep G2 cells ectopically co-expressing FLAG-BSEP, mCherry-CHMP5 and either HA-Ub-K63 or HA-Ub-K63R for 12, 18, and 24 h. In Ub-K63 expressing cells, FLAG-BSEP signals were detected earlier and more abundantly in the PM fractions compared with that in the Ub-K63R expressing cells (Fig. 5b). In line with the cell-based results (Fig. 5b), K63-linked ubiquitination being able to influence BSEP apical-targeting was also revealed in the hydrodynamic injection-based mouse model. As shown in Fig. 5c, more canalicular FLAG-BSEP staining was observed in mouse livers with Ub-K63 co-expression in comparison with those with Ub-K63R co-expression. Temperature shift assays combined with FLAG-BSEP staining were used to study the influence of Ub-K63 and CHMP5 on post-Golgi trafficking of BSEP in Hep G2 cells (Fig. 5d). In Ub-K63R co-expressing cells, FLAG-BSEP staining on the plasma membrane was hardly detected. On the other hand, FLAG-BSEP signals could be detected on the plasma membrane in Ub-K63 expressing Hep G2 pre-treated with si-CTL, but reduced in CHMP5 depleted cells (Fig. 5d). These data suggested that K63-linked ubiquitination of BSEP is required for CHMP5-mediated BSEP membrane targeting.

### The ESCRT machinery affects the polarized trafficking of BSEP

VPS4, the only ESCRT molecule harboring enzyme activity, dissembles and recycles ESCRT-III complexes to complete ESCRT involved processes [25]. CHMP5 and other ESCRT-III proteins could recruit and regulate VPS4 through LIP5 [26, 27]. Mammalian cells express two non-allelic VPS4 paralogs, VPS4A and VPS4B [28]. We co-expressed FLAG-BSEP and the mCherry-tagged, wild type VPS4 (mCherry-VPS4A or mCherry-VPS4B) or its dominant negative mutant (mCherry-VPS4A-E228Q [mCherry-VPS4A-EQ] or mCherry-VPS4B-E235Q [mCherry-VPS4B-EQ]) to investigate whether the ESCRT machinery affected BSEP trafficking in Hep G2 cells via temperature shift assay. As shown in Additional file 1: Figure S6A, the abundance of FLAG-BSEP in the PM fraction was reduced in the cells co-expressing VPS4A-EQ in comparison with the cells co-expressing wild-type VPS4A after release from the Golgi temperature block. Similarly, dominant negative mutant mCherry-VPS4B-EQ, in contrast to mCherry-VPS4B, also impaired post-Golgi trafficking of BSEP (Additional file 1: Figure S6B). These results inferred that the ESCRT machinery indeed affected the post-Golgi-trafficking of BSEP.

### Either VPS4A or VPS4B is upstream of Rab11A to affect the post-Golgi trafficking of BSEP

**Fig. 6:**
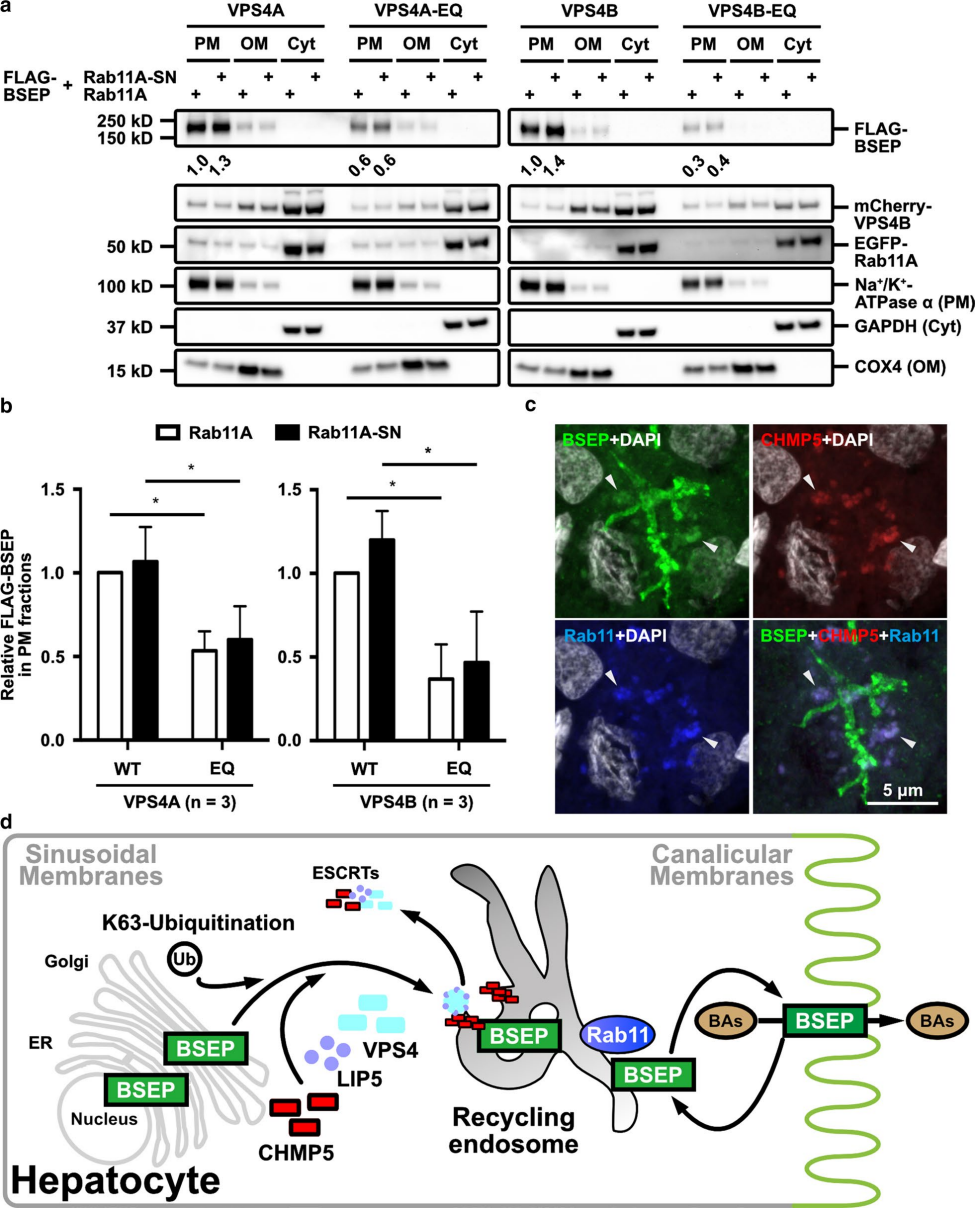
ESCRTs affecting apical targeting of BSEP is upstream of Rab11.** a** VPS4-mediated the post-Golgi trafficking of BSEP was upstream of Rab11A. Hep G2 cells were co-expressed the indicated combination of FLAG-BSEP; EGFP-Rab11A or EGFP-Rab11A-S25N (EGFP-Rab11A-SN); mCherry-VPS4A or -VPS4A-E228Q (VPS4A-EQ); or mCherry-VPS4B or -VPS4B-E235Q (VPS4B-EQ) for temperature shift assay. A representative immunoblotting reveals the plasma-membrane (PM), organelle-membrane (OM) and cytosolic (Cyt) protein fractions detected by the indicated proteins and the fractionation controls. These experiments, described in (**a**), were independently triplicated. The values are the relative FLAG-BSEP signals normalized to the corresponding fractionation control signals. The independently triplicated results (n = 3, mean ± SD) were illustrated in (**b**). **c** Confocal microscopy demonstrates the BSEP-and-CHMP5 colocalized SACs are also positive with the recycling endosome marker Rab11 (arrowhead). Adult human liver samples were co-immunofluorescently stained for BSEP (green), CHMP5 (red), and Rab11 (blue pseudo-color). DAPI (white pseudo-color) stained the nuclei of hepatocytes. **d** After transcription and translation, BSEP undergoes core-and complete-glycosylation in the ER and Golgi, respectively, and subsequent post-Golgi trafficking. BSEP is poly-ubiquitinated via K63 linkages and bound with CHMP5 further targets to the sorting endosomes. The sorting endosome is an internal trafficking hub enriched with the small GTPase Rab5 proteins. Other ESCRT molecules, at least LIP5, may co-localize with BSEP and CHMP5 at the sorting endosome. VPS4 dissembles ESCRTs on the sorting endosomes, from which BSEP is transferred to the canalicular membrane via Rab11-mediated targeting and then cycles between the Rab11-recycling endosomes

Post-Golgi trafficking of BSEP has been known to pass through the endosomal intermediates that are regulated by small GTPases such as Rab5 (the early/sorting endosome) and Rab11 (the recycling endosome) [10, 29–34]. At steady state, BSEP constitutively cycled between Rab11-positive compartments and the canalicular membrane in polarized hepatic cells [10, 33]. Moreover, ESCRTs molecules have been referred to co-localize with Rab proteins at endosomal compartments [35]. The BSEP trafficking regulated by ESCRTs being up- or down-stream of Rab11 was unknown. To address this question, we co-expressed mCherry-VPS4 or mCherry-VPS4-EQ together with either EGFP-Rab11A or dominant negative EGFP-Rab11A-S25N (EGFP-Rab11A-SN) in cells, which were followed by temperature shift assays and subcellular fractionation. In the cells expressing dominant negative VPS4 proteins, the FLAG-BSEP signals in PM fractions, regardless of wild type or dominant negative Rab11A co-expression, were significantly reduced in comparison of the wild-type VPS4 proteins expressing cells (Fig. 6a, b). Therefore, ESCRTs affecting BSEP trafficking was upstream of Rab11A.

We wondered whether those BSEP-and-CHMP5 double positive SACs observed in adult human livers (Fig. 2e) were also Rab11 positive. The distribution of BSEP, CHMP5 and Rab11 in adult human livers were co-immunofluorescently stained and analyzed through confocal microscopy. As shown in Fig. 6c, BSEP-and-CHMP5 positive SACs in human hepatocytes are also with Rab11 signals, suggesting BSEP-resident SACs are the ESCRT-and-Rab11 co-localized endosome.

## Discussion

BSEP is the key bile salt transporter in the canalicular membrane of hepatocytes. BSEP abnormalities result in different degrees of cholestasis and hepatocellular injuries. Defects in BSEP function and canalicular expression are also pivotal events that have caused the failure of many newly developed drugs [36]. Since the discovery of HAX-1 being a BSEP interacting protein, the internalization mechanism of BSEP has been extensively studied [37–42]. On the other hand, the mechanism of post-Golgi targeting of BSEP to apical/canalicular membrane has not been fully characterized so far. Besides, whether ESCRTs participate in the post-Golgi sorting of apical membrane proteins, such as BSEP, has not been explored.

In this study, our results show the first example that ESCRT-III is essential for polarized trafficking of the apical membrane protein BSEP. The ESCRT-III subunits CHMP5 and LIP5 co-localized with BSEP in hepatocytes at the SACs, which were further evidenced to be the Rab11-positive endosomes. Defective canalicular sorting of BSEP in cholestatic human livers was accompanied by aberrant morphology of subapical BSEP vesicles that were CHMP5 positive. BSEP mutants with altered affinity to CHMP5, such as R487H or N490D, may cause the intracellular retention and defective apical expression, and lead to cholestatic diseases. Both CHMP5 and VPS4 of the ESCRT-III machinery affected the post-Golgi trafficking of BSEP, which was upstream of Rab11A-mediated BSEP sorting. ESCRT-III regulated BSEP targeting required BSEP to be ubiquitinated via K63 linkages. Taken together, CHMP5 indirectly influenced BSEP-mediated bile salt secretion. Human liver samples at different stages demonstrated the association and co-localization of BSEP with CHMP5 in the cytoplasm, indicating the canalicular trafficking of BSEP is developmentally regulated and possibly mediated through CHMP5-associated ESCRT-III machinery.

The ESCRT machinery is known to be necessary for membrane remodeling that includes multivesicular body (MVB) formation; cell polarity establishment; exosome biosynthesis; enveloped virus budding; nuclear and plasma membrane repair; autophagy biogenesis and cytokinesis [23, 43]. Recently, some ESCRT molecules have been shown to participate in the unconventional protein secretion of the cystic fibrosis conductance transmembrane regulator (CFTR) mutant [44]. Whether ESCRTs participate in the post-Golgi trafficking of BSEP as well as other apical proteins is so far unknown. Interestingly, both BSEP and CHMP5 have been identified in human urinary exosomes, implying that BSEP could be an ESCRT cargo [45].

In contrast to sparse information on ESCRTs functioning on polarized sorting, a significant body of data suggested apical and basolateral membrane proteins traffic through Rab-associated endosomal compartments to target the destinated membrane domain in polarized cells [29, 46–50]. Studies in rodents [8, 29] and polarized WIF-B9 cell models [10, 33] have demonstrated the apical targeting of BSEP traversed Rab11-positive endosomal compartments. Rab11 locates at the *trans*-Golgi, post-Golgi vesicles, sorting/early endosome (EE), and recycling endosomes. Together with Rab11 interacting proteins and effectors, Rab11 regulates both the secretory pathway and endosomal recycling [32]. Bsep and Bsep mutants were shown to localize at Rab11-positive compartments in cultured cell lines [10, 33, 51]. Mutations in Rab11 interacting proteins [52] or effectors [53, 54] have been known to cause cholestasis and BSEP mis-sorting. In addition to the ESCRT-III proteins CHMP5 and LIP5, we also observed the co-localization of intracellular BSEP and Rab11-positive compartments in co-transfected Hep G2 cells (data not shown) and in adult human liver samples.

Our data revealed the dominant-negative VPS4 affecting BSEP apical sorting overriding Rab11A influences. Dominant-negative VPS4 has been reported to inhibit the early endosomal recycling of several proteins and cholesterol to either the plasma membrane or the *trans*-Golgi network (TGN) in mammalian cells [55, 56]. Moreover, the localization of ESCRTs at the TGN/EE has been observed in plant models, and the dominant-negative ESCRT-III protein VPS2 affected the TGN/EE and perturbed the subsequent sorting [57]. These findings suggested that ESCRTs functioning on cargo sorting precede the early/sorting endosomes. Notably, hepatocyte-specific knockdown of the early endosomal protein Rab 5 in mice caused Bsep cytoplasmic retention together with a significant reduction (~ 30% of the control) in canalicular distribution [29]. Hence, ESCRTs possibly affected BSEP trafficking on the route from the TGN to the sorting endosome, from which BSEP entered Rab11-regulated compartments. The increased association between ESCRTs and BSEP mutants (such as R487H) may perturb the release of BSEP mutants from the sorting endosome, which may partially explain the targeting defect and disease mechanism of cholestasis in patients bearing these BSEP mutants [12, 14, 58–60].

Combined with our findings and the results in the literature, we proposed a model of the ESCRT machinery in the apical targeting of BSEP (Fig. 6d). After post-translational modification, newly synthesized BSEP undergoes post-Golgi trafficking through vesicle carriers to target the canalicular membrane of hepatocytes. BSEP is modified with K63-linked polyubiquitin chains and transported via CHMP5-associated ESCRT machinery to the Rab11-positive recycling endosome. VPS4 is recruited to these vesicles and dissociates CHMP5-and-LIP5 bound ESCRT complexes from BSEP. BSEP enters the Rab11-regulated transport to target canalicular membrane, on which BSEP mediates bile salt secretion. Finally, canalicular BSEP undergoes the Rab11-regulated apical cycling [10]

## Conclusions

We provided evidence that the ESCRT machinery mediates apical targeting of the canalicular transporter BSEP. Dysregulated ESCRT functions may result in subapical BSEP retention and cholestasis. As the mechanism of BSEP targeting concerning bile physiology and cholestatic diseases have been more elucidated, the finding of the novel BSEP-interacting protein CHMP5 and the unraveling of the mechanism mediating the polarized trafficking of BSEP via ESCRT machinery may provide clues for developing new therapeutic approaches for BSEP-associated cholestasis.

## Supplementary Information

**Additional file 1: Figure S1.** BSEP is retained at aberrant CHMP5-positive subapical compartments in a transient cholestatic human liver sample. **Figure S2.** The total membrane-protein fraction contains the plasma-membrane plus organelle-membrane protein fractions. **Figure S3.** The ESCRT-III subunits CHMP5 and LIP5 co-localize with BSEP-resident subapical compartments in adult human hepatocytes. **Figure S4.** The canalicular targeting of BSEP is developmentally regulated and associated with CHMP5 in human livers. **Figure S5.** The protein expression and turnover of BSEP is unaffected with CHMP5 knockdown. **Figure S6.** Both VPS4A and VPS4B affect post-Golgi trafficking of BSEP.

**Additional file 2: Table S1.** Classification of the cDNA inserts identified from the yeast two-hybrid screen for BSEP-interacting proteins

## Data Availability

All data generated or analyzed during the current study are available from the corresponding author on reasonable request.

## Abbreviations

BRIC2: Benign recurrent intrahepatic cholestasis type 2
BSEP: Bile salt export pump
CFTR: Cystic fibrosis conductance transmembrane regulator
CHMP5: Charged multivesicular body protein 5
CTL: Control
Cyt: Cytosolic fraction
EE: Early endosome
EGFP: Enhanced green fluorescence protein
ESCRT: Endosomal protein complex required for transport
HA: Hemagglutinin
MVB: Multivesicular body
NBF: Nucleotide-binding fold
tM: Total membrane-protein fraction
OM: Organelle-membrane protein fraction
PFIC2: Progressive familial intrahepatic cholestasis type 2
PM: Plasma membrane protein fraction
SAC: Subapical compartment
shRNA: Small-hairpin RNA
siRNA: Small-interference RNA
TGN: *Trans*-Golgi network
Ub: Ubiquitin
Wt: Wild type
