# Supplementary material for: The ESCRT-III molecules regulate the apical targeting of bile salt export pump
Source: J Biomed Sci. 2021 Mar 9;28:19. doi: 10.1186/s12929-020-00706-2 (PMC7941988; doi:10.1186/s12929-020-00706-2)
Supplement: Supplementary file 1 — Additional file 1: Figure S1. BSEP is retained at aberrant CHMP5-positive subapical compartments in a transient cholestatic human liver sample. Figure S2. The total membrane-protein fraction contains the plasma-membrane plus organelle-membrane protein fractions. Figure S3. The ESCRT-III subunits CHMP5 and LIP5 co-localize with BSEP-resident subapical compartments in adult human hepatocytes. Figure S4. The canalicular targeting of BSEP is developmentally regulated and associated with CHMP5 in human livers. Figure S5. The protein expression and turnover of BSEP is unaffected with CHMP5 knockdown. Figure S6. Both VPS4A and VPS4B affect post-Golgi trafficking of BSEP. [file 12929_2020_706_MOESM1_ESM.pdf]

The ESCRT-III Molecules Regulate the Apical Targeting of Bile Salt Export Pump

Shang-Hsin Wu[1], Mei-Hwei Chang[1, 2, 3], Hui-Ling Chen[3], Hui-Lin Wu[1,3], Huey-Huey Chua[2], Chin-Sung Chien[1], Ya-Hui Chen[2], Yen-Hsuan Ni[2, 3, 4], Huey-Ling Chen[2, 3, 5, 6, *]

Figure S1. BSEP is retained at aberrant CHMP5-positive subapical compartments in a transient cholestatic human liver sample.

Figure S2. The total membrane-protein fraction contains the plasma-membrane plus organelle-membrane protein fractions.

Figure S3. The ESCRT-III subunits CHMP5 and LIP5 co-localize with BSEP-resident subapical compartments in adult human hepatocytes.

Figure S4. The canalicular targeting of BSEP is developmentally regulated and associated with CHMP5 in human livers.

Figure S5. The protein expression and turnover of BSEP is unaffected with CHMP5 knockdown.

Figure S6. Both VPS4A and VPS4B affect post-Golgi trafficking of BSEP.

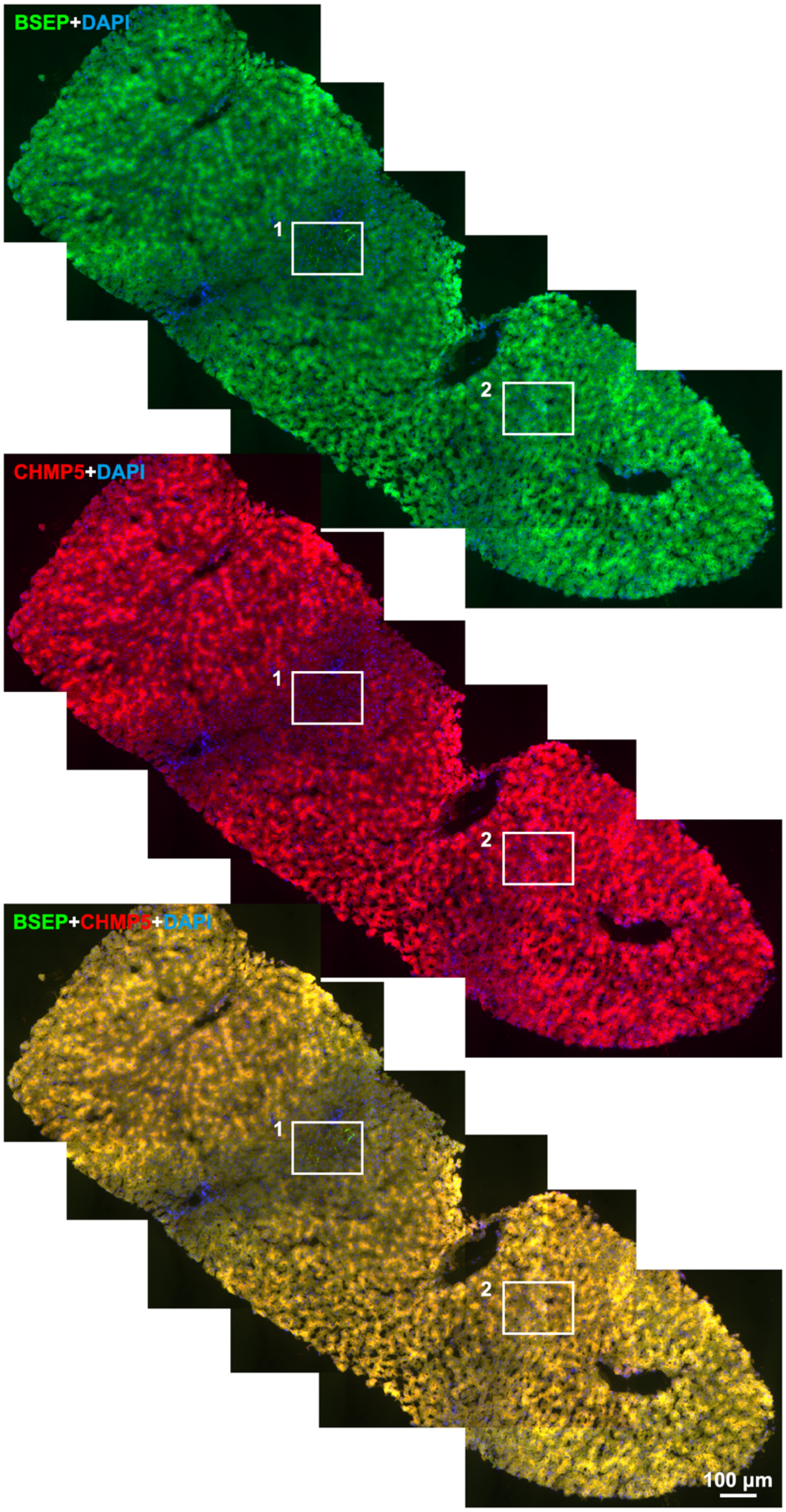

BSEP+DAPI
1
2
CHMP5+DAPI
1
2
BSEP+CHMP5+DAPI
1
2
100 µm

Figure S1. BSEP is retained at aberrant CHMP5-positive subapical compartments in a transient cholestatic human liver sample.

Immunofluorescence staining demonstrated the co-localization of subapical BSEP and CHMP5 in the cytoplasm of hepatocytes in a transient cholestatic liver sample. Cryosections of the human liver were immunofluorescently labeled with BSEP (green) and CHMP5 (red). The liver cell nuclei were stained with DAPI. Notably, a small region of hepatocytes reveals canalicular BSEP, but others show aggregated signals of BSEP and CHMP5 in the cytoplasm. The 200x images were manually integrated using the program Adobe Photoshop CS6. The white rectangles numbered are the similar area of the 400x images shown in **Figure 1E** (area 1 and area 2).

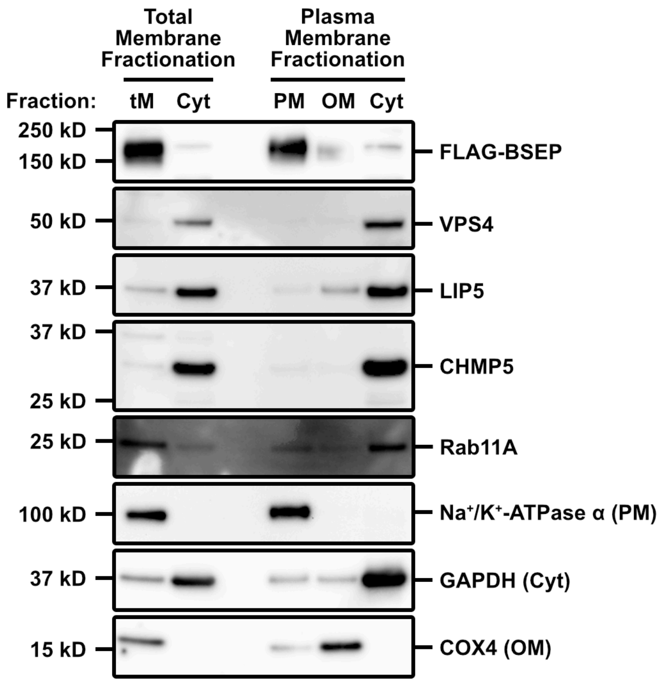

Figure S2. The total membrane-protein fraction contains the plasma-membrane plus organelle-membrane protein fractions.

Hep G2 cells were transfected with p3XFLAG-BSEP and then fractionated through two different fractionation methods. One is to isolate the total membrane-protein (tM) and the cytosolic (Cyt) fractions. The other is to fractionated into the plasma-membrane (PM), the organelle-membrane (OM), the cytosolic (Cyt) and the nuclear protein fractions. Each protein was probed via indicated antibodies. Na^+/K^+-ATPase α, COX4, and GAPDH were used as a PM, an OM, and a Cyt fractionation controls.

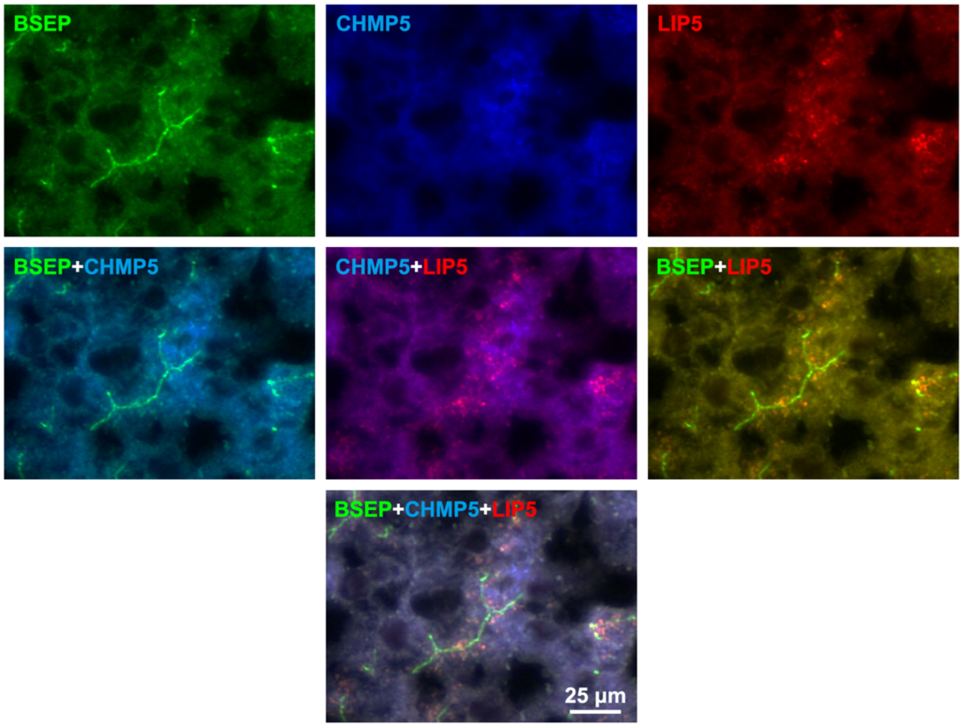

Figure S3. The ESCRT-III subunits CHMP5 and LIP5 co-localize with BSEP-resident subapical compartments in adult human hepatocytes.

Cryosections of the adult human livers were co-immunofluorescently stained for BSEP (green), CHMP5 (blue), and the CHMP5-interacting protein LIP5 (red).

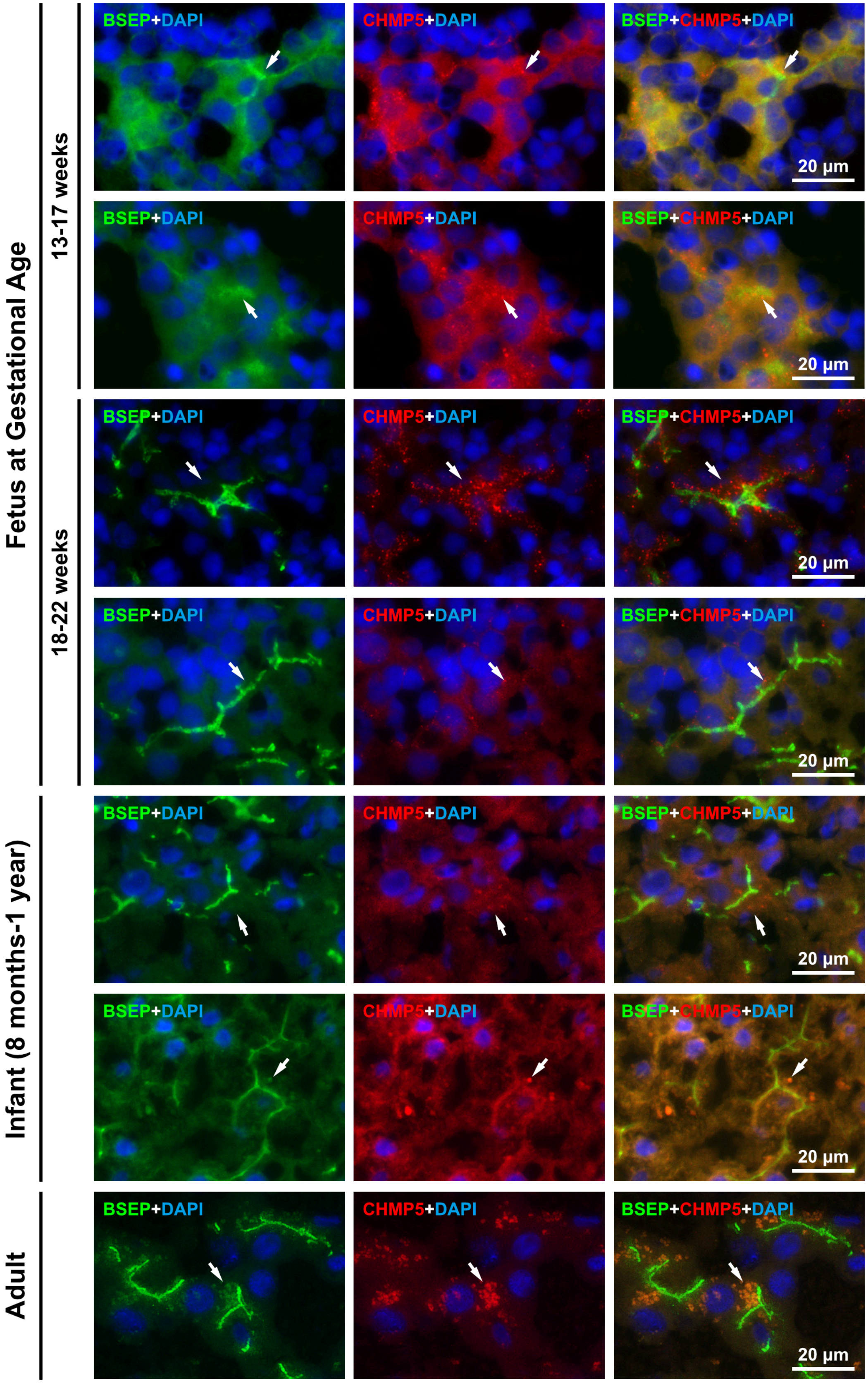
Fetus at Gestational Age
13-17 weeks
18-22 weeks
Infant (8 months-1 year)
Adult
BSEP+DAPI
CHMP5+DAPI
BSEP+CHMP5+DAPI
20 µm

Figure S4. The canalicular targeting of BSEP is developmentally regulated and associated with CHMP5 in human livers.

Immunofluorescence staining demonstrated the distribution of BSEP and CHMP5 in the human liver samples from fetus at gestational age 13-22 weeks (n = 21), infant at 8 months-1year (n = 4), and adult control (n = 3). Cryosection of human livers samples were immunostained for BSEP (green) and CHMP5 (red). The liver nuclei were stained with DAPI. Arrows indicate the different degree of CHMP5 that co-localizes with subapical BSEP.

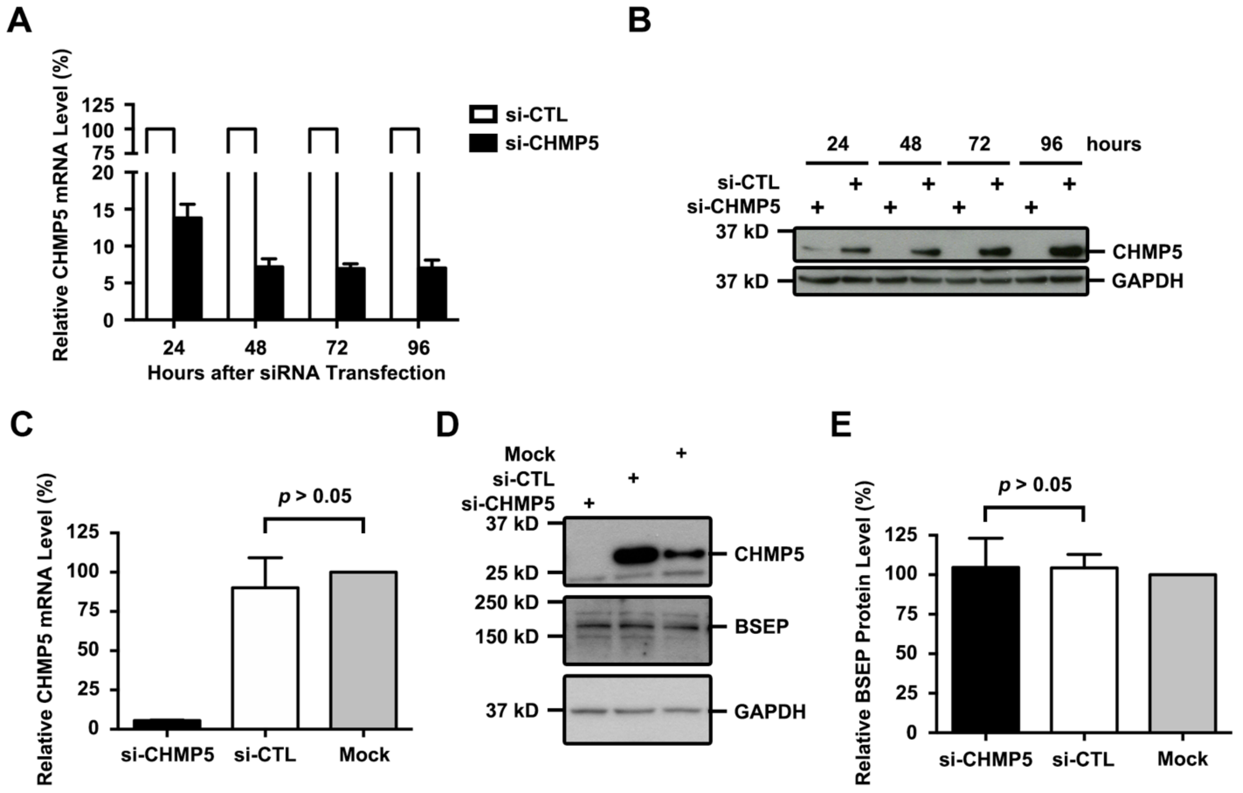

Figure S5. The protein expression and turnover of BSEP is unaffected with CHMP5 knockdown.

(A and **B)** Endogenous CHMP5 in Hep G2 cells was knocked down and sustained in steady state by small interference RNA (siRNA) in 48 hours. Hep G2 cells were reverse transfected with 10 nM of a *CHMP5*-targeting (si-CHMP5) or a non-targeting (si-CTL) pools for the indicated times. **(A)** The Bar graph (n = 3, mean ± SD) demonstrates the quantitative PCR results of the relative *CHMP5* mRNA levels, which were normalized to *GAPDH*, and the si-CTL group at each corresponding time point was defined as 100%. **(B)** Representative immunoblots of CHMP5 and GAPDH demonstrate CHMP5 knockdown at the protein level. The protein GAPDH was used as a loading control. **(C** and **D)** Hep G2 cells were reverse transfected with si-CHMP5 or si-CTL for 48 hours. **(C)** The Bar graph (n = 3, mean ± SD) demonstrates the quantitative PCR result of the relative *CHMP5* mRNA level, which were normalized to *gapdh*. The Mock group was defined as

100%. **(D)** Representative immunoblot of BSEP, CHMP5 and GAPDH demonstrates CHMP5 knockdown and BSEP at the protein level. GAPDH was used as a loading control. **(E)** The bar graph (n = 3, mean ± SD) illustrates the relative BSEP protein levels, which were unaffected by *CHMP5* knockdown. Densitometry was applied to quantify the signal densities of BSEP and GAPDH from **(D).** The Mock group was defined as 100%. The *P*-value was calculated by *Student's t*-test.

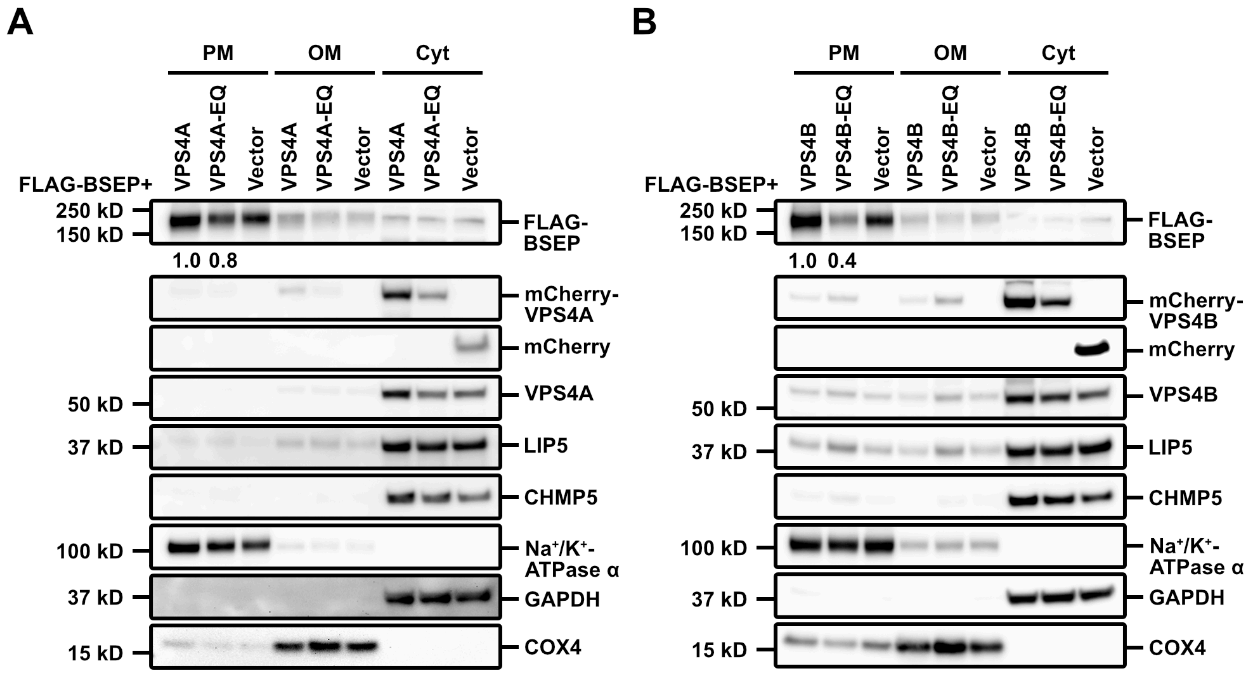

Figure S6. Both VPS4A and VPS4B affect post-Golgi trafficking of BSEP.

Disturbed membrane targeting of BSEP in dominant-negative VPS4A or VPS4B mutant co-expressing cells was revealed through temperature shift assay followed by subcellular fraction and immunoblotting. Hep G2 cells co-expressing FLAG-BSEP and either mCherry-tagged **(A)** VPS4A, VPS4A-E228Q (VPS4A-EQ), **(B)** VPS4B, or VPS4B-E235Q (VPS4B-EQ) were subjected to temperature shift assay. VPS4A-EQ and VPS4B-EQ are the dominant negative VPS4A and VPS4B mutants, respectively. The plasmid pmCherry-C1 was used as the vector control. A representative immunoblotting reveals the plasma-membrane (PM), organelle-membrane (OM) and cytosolic (Cyt) protein fractions detected by the indicated protein antibodies. Na^+/K^+-ATPase α, COX4 and GAPDH were used as a PM, an OM and a Cyt fractionation controls. The values are the relative FLAG-BSEP signal normalized to the corresponding fractionation control signal.
