# Supplementary material for: The ESCRT-III molecules regulate the apical targeting of bile salt export pump
Source: J Biomed Sci. 2021 Mar 9;28:19. doi: 10.1186/s12929-020-00706-2 (PMC7941988; doi:10.1186/s12929-020-00706-2)
Supplement: Supplementary file 2 — Additional file 2: Table S1. Classification of the cDNA inserts identified from the yeast two-hybrid screen for BSEP-interacting proteins [file 12929_2020_706_MOESM2_ESM.pdf]

# The ESCRT-III Molecules Regulate the Apical Targeting of Bile Salt Export Pump

**Shang-Hsin Wu[1], Mei-Hwei Chang[1, 2, 3], Hui-Ling Chen[3], Hui-Lin Wu[1,3], Huey-Huey Chua[2], Chin-Sung Chien[1], Ya-Hui Chen[2], Yen-Hsuan Ni[2, 3, 4], Huey-Ling Chen[2, 3, 5, 6, *]**

**Table S1. 1 Classification of the cDNA inserts identified from the yeast two-hybrid screen for BSEP-interacting proteins.**

**Table S1. 1 Classification of the cDNA inserts identified from the yeast two-hybrid screen for BSEP-interacting proteins**

| Functions | cDNA identity | Levels* of β-gal |
|---|---|---|
| Protease inhibition | Serpin peptidase inhibitor clade A member 1 (SERPINA1, anti-trypsin) | 2, 3 |
| | Serpin peptidase inhibitor clade A member 3 (SERPINA3, anti-chymotrypsin) | 3 |
| | Alpha-1-microglobulin/bikunin precursor (AMBP) | 3 |
| Protein sorting | Charged multivesicular body protein 5 (CHMP5) | 2 |
| | Coatomer protein complex, subunit alpha (COPA) | 3 |
| Glycosylation | Hexokinase domain containing 1 (HKDC1), mRNA | 2, 3 |
| | Proteoglycan 4 (PRG4) | 3 |
| Cytoskeleton remodeling | Cofilin 1 (non-muscle) (CFL1), mRNA | 3 |
| Protein folding and degradation | DnaJ (Hsp40) homolog, subfamily C, member 9 | 3 |
| | DDB1 and CUL4 associated factor 11 (DCAF11), transcript variant 5, non-coding RNA | 2 |
| | Proteasome subunit beta type 6 (PSMB6), mRNA | 3 |
| | Proteasome subunit beta type, 10 (PSMB10) | 2 |
| | Amyloid beta (A4) precursor-like protein 2 (APLP2) | 2 |
| Metabolism | Farnesyl-diphosphate farnesyltransferase 1 (FDFT1), mRNA | 3 |
| | Inter-alpha-trypsin inhibitor heavy chain 2 (ITIH2) | 2 |
| Physical homeostasis | Albumin (ALB) | 2, 3 |
| | Hemoglobin, alpha 1 (HBA1) | 2, 3 |
| | Hemoglobin, gamma A (HBG1), mRNA | 2 |
| Chromatin modification | Centromere protein A (CENPA) | 1 |
| | H2A histone family, member J (H2AFJ) | 2, 3 |
| | H3 histone, family 3A, pseudogene 4 (H3F3AP4) | 2, 3 |
| | H3 histone, family 3B (H3F3B) | 2, 3 |
| | HIST1H2AC histone cluster 1 (H2ac) | 2, 3 |
| | HIST1H2BG histone cluster 1, (H2bg) | 2, 3 |
| | HIST1H2BK histone cluster 1, (H2bk) | 2, 3 |
| | HIST1H2BD transcript variant 1, mRNA | 2, 3 |
| | HIST1H2BG, mRNA | 2, 3 |
| | HIST2H2BE, mRNA | 2, 3 |
| | HIRA interacting protein 3 (HIRIP3), | 2, 3 |
| | SET domain containing 1A (SETD1A) | 2, 3 |
| | Homologous Alu RNA binding protein (SRP14) | 2, 3 |
| | X-ray repair complementing defective repair in Chinese hamster cells 6 (XRCC6) | 2, 3 |
| | Zinc finger and BTB domain containing 16 (ZBTB16) | 2, 3 |
| Transcription, translation, or mRNA processing | MT-RNR2-like 8 (MTRNR2L8) | 2 |
| | POLM polymerase (DNA directed), mu | 3 |
| | Serine/arginine-rich splicing factor 4 (SRSF4), mRNA | 2 |

| | | |
|---|---|---|
| Others | Mitochondrion, complete genome | 2 |

*The levels 1, 2, and 3 of β-gal represent the permeabilized yeasts with blue appearance in 0.5, 0.5-to-1, and 1-to-2 hours, respectively, after X-gal incubation.
